# Hepatitis B virus-targeting sodium taurocholate cotransporting polypeptide mediates HBV infection and damage in human renal podocytes

**DOI:** 10.1128/spectrum.01365-23

**Published:** 2024-02-05

**Authors:** Lifen Wang, Cheng Wang, Xu Wang, Yantao Cao, Xiaohua Guo, Zhiming Ye

**Affiliations:** 1 Department of Nephrology, Shenzhen Hospital of Southern Medical University, Shenzhen, China; 2 Department of Nephrology, Guangdong Academy of Medical Sciences, Guangdong Provincial People's Hospital, Guangzhou, China; University of Arizona, Tucson, Arizona, USA

**Keywords:** NTCP, podocytes, hepatitis B virus, glomerulonephritis

## Abstract

Hepatitis B virus (HBV) may directly infect human podocytes (HPCs). However, the mechanism of direct infection is unclear. We found that HPCs express sodium taurocholate cotransporting polypeptide (NTCP), a specific receptor for HBV entry into hepatocytes. Thus, we investigated whether NTCP mediates HBV infection and damage in HPCs and further clarified the specific mechanism. We constructed shRNA-NTCP1,2, shRNA-NC, WT-NTCP, and MUT-NTCP and transfected them into HPCs. HPCs were infected with HBV, and HBV infection markers were detected by enzyme-linked immunosorbent assay (ELISA) and real-time quantitative PCR (RT-qPCR). The functional changes in HPCs were detected by Transwell migration and scratch assays, apoptosis was evaluated by flow cytometry (FCM), and podocytoskeletal proteins (nephrin, CD2AP, and synaptopodin) were determined by western blotting (WB). Compared with the control HPCs, HPCs infected with HBV showed increased levels of HBV infection markers and apoptosis along with decreased podocytoskeletal protein expressions, cell vitality, proliferation, and migration. Compared with the HPCs infected with HBV, the HPCs transfected with HBV + shRNA-NTCP, and HBV + MUT-NTCP showed decreased levels of HBV infection markers and apoptosis along with increased podocytoskeletal protein expressions, cell vitality, proliferation, and migration; the opposite effects were observed in the HPCs transfected with HBV + WT-NTCP. Overall, the changes to NTCP affected the susceptibility of HPCs to HBV and modulated HPC damage and repair. NTCP can mediate direct HBV infection and damage human podocytes, and the NTCP 157–165 locus is the main site of HBV entry. The findings provide a new target and theoretical basis for HBV-associated glomerulonephritis.

**IMPORTANCE:**

This study identified for the first time that sodium taurocholate cotransporting polypeptide (NTCP) can mediate HBV direct infection and damage to human podocytes, and the NTCP157–165 locus is the main HBV entry site. The findings provide theoretical support for the pathogenesis of direct infection of HBV with kidney tissue. The findings provide a new target and theoretical basis for the treatment of HBV-related glomerulonephritis (HBV-GN). Blocking NTCP is a new target for the treatment of HBV-GN. We found that tacrolimus, a calcineurin inhibitor that blocks NTCP, can effectively treat HBV-GN. This study also provides a theoretical basis for the effective and safe treatment of immunosuppressant tacrolimus for HBV-GN.

## INTRODUCTION

Chronic hepatitis B virus (HBV) infection mainly involves the liver. HBV is associated with mild pancytopenia and can lead to extrahepatic lesions; 16% of patients with chronic HBV infection develop extrahepatic manifestations of diseases such as nephritis and Sjogren’s syndrome, of which 3% of cases are attributed to HBV (1, 2). Currently, the pathogenesis of HBV-related glomerulonephritis (HBV-GN) mainly includes the following: (i) humoral immune-mediated injury, primarily caused by the deposition of circulating and *in situ* immune complexes (3, 4); (ii) cellular immune mechanisms (5); (iii) the induction of a variety of autoantibodies by HBV, resulting in autoimmune damage (6); and (iv) the direct HBV infection of renal tissue [integrated and free HBV DNA and HBV cccDNA, a replication intermediate, have been detected in renal tissue (7), suggesting that HBV may directly infect renal tissue]. However, the mechanism of HBV infection in glomerular podocytes remains unclear.

In 2012, Wenhui et al. found that sodium taurocholate cotransport polypeptide (NTCP), an HBV-specific receptor and hepatic bile acid salt transporter, expresses on human hepatocyte membranes (8). NTCP binds specifically to the PreS1 region of the large envelope protein (L protein) of HBV to mediate HBV entry into hepatocytes, after which HBV replication occurs in hepatocytes (9, 10). A series of experiments performed in human primary hepatocytes and hepatocellular carcinoma cells confirmed that hepatic NTCP is the true cellular receptor required for HBV to enter hepatocytes (8). The Pres1 region of the L protein of HBV interacts with amino acids at sites 157–165 of NTCP; HBV is then internalized into hepatocytes, causing massive virus replication (8, 11). Human NTCP has been shown to play an important role in the entry of HBV into cells and subsequent infection. Thus, blocking human NTCP has become a new target for the treatment of HBV infection (12–16).

Our previous experiments confirmed the expression of NTCP in human glomerular podocytes. However, it was not clear whether HBV bound to NTCP in the cytosolic membranes of human glomerular podocytes would enter and infect the podocytes and further damage them. We perform experiments to investigate the association of podocyte and HBV infection. Human NTCP-overexpressing and knock-down constructs up- and downregulate the expression of NTCP receptor in cultured human podocytes. The HBV used in this study was mainly derived from the culture supernatant of Hepg2.2.15 cells. HBV infected the cultured human podocytes. We detected the marker of HBV infection in podocytes. Meanwhile, the proliferation, apoptosis, and function of podocytes were detected. In addition, after the NTCP of the podocytes was downregulated by shRNA-NTCP, we observed that HBV infection and function changed in podocytes. To investigate the interaction of HBV and NTCP, we further mutated amino acids 157–165 of the human podocyte NTCP. The study is expected to reveal the new pathogenesis of HBV-infected and -injured podocytes, which will provide new target and theory bases for the treatment of HBV-GN.

## RESULTS

### HBV successfully infected HPCs

**Fig 1:**
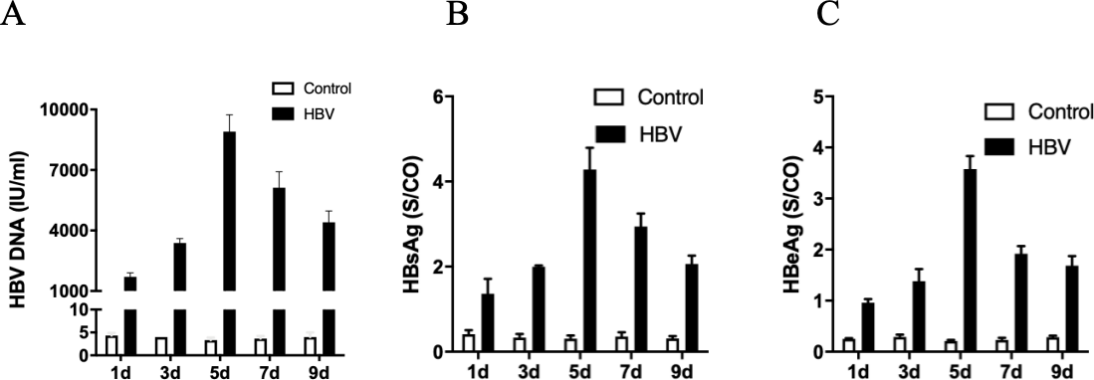
HBV-infected human podocytes. Differentiated HPCs were incubated with HepG 2.2.15 supernatant (containing HBV) for 16 h. The free culture supernatant was then discarded to remove HBV from the culture supernatant, and the culturing was continued until days 1, 3, 5, 7, and 9 after infection. The levels of HBV DNA (A) in the supernatant from the indicated groups were measured by quantitative PCR. The levels of HBV DNA peaked on day 5 of infection (A). The levels of HBsAg (B) and HBeAg (C) in the supernatant from the indicated groups were detected by enzyme-linked immunosorbent assay. The levels of HBsAg peaked on day 5 of infection (B). The levels of HBeAg peaked on day 5 of infection (C). Results are mean ± SD for three individual experiments.

To confirm that human podocytes (HPCs) expressing NTCP can be infected by HBV, we used HBV-containing supernatants from Hepg2.2.15 cells to infect HPCs. The levels of HBV DNA, hepatitis B surface antigen (HBsAg), and hepatitis B e-antigen (HBeAg) were significantly higher in the podocytes + HBV (HBV) group compared to the podocytes (control) group (Fig. 1A through C).There were no significant changes in the HBV DNA, HBsAg, and HBeAg levels in the control group over time. In contrast, the levels of HBV DNA, HBsAg, and HBeAg in the HBV group increased over time and peaked on day 5, after which they began to decrease (Fig. 1A through C). Thus, subsequent experiments were conducted for 5 days after infection.

### shRNA mediated the knockdown of hNTCP to eliminate HPC susceptibility to HBV

**Fig 2:**
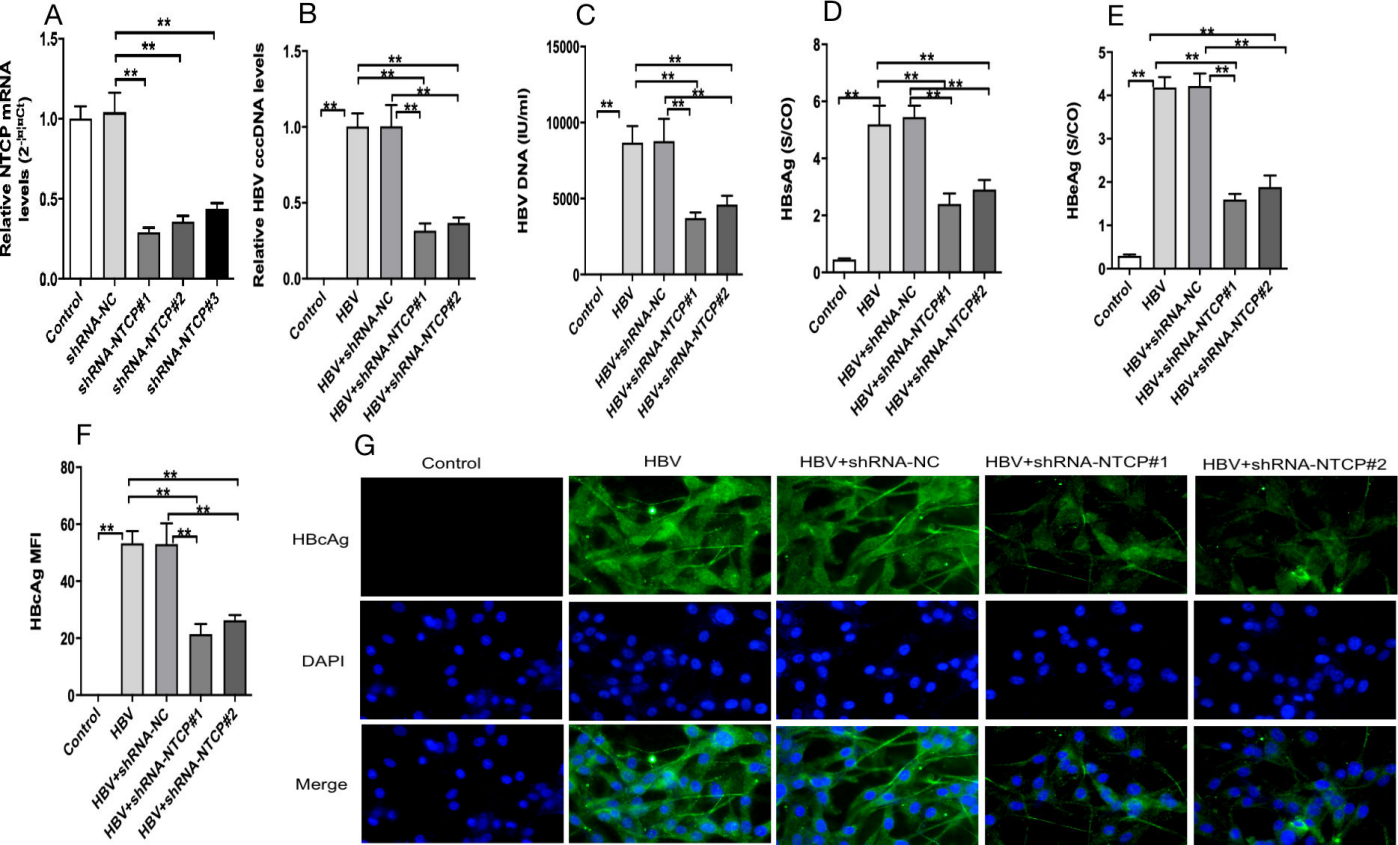
Effect of NTCP downregulation and NTCP blockade on HBV-infected HPCs. Differentiated podocytes were transfected with shRNA-NC, shRNA-NTCP#1, or shRNA-NTCP#2 by lentiviral plasmid, and the expression of NTCP measured by qPCR was significantly downregulated after transfection with shRNA-NTCP (**A**) HPCs in the HBV, HBV + shRNA-NC (a control plasmid), shRNA-NTCP#1, and shRNA-NTCP#2 groups were incubated with HepG 2.2.15 supernatant (containing HBV) for 16 h, with HPCs incubated with cell culture medium as the control. The levels of HBV cccDNA (**B**) and HBV DNA (**C**) were measured by qPCR. The levels of HBsAg (**D**) and HBeAg (**E**) in the supernatant from the indicated groups were detected by enzyme-linked immunosorbent assay. (**F, G**) Representative images of HBcAg (green) and 4′,6-diamidino-2-phenylindole (DAPI) nuclear (blue) staining in differentiated human podocytes. The levels of HBV cccDNA (**B**), HBV DNA (**C**), HBsAg (**D**), HBeAg (**E**), and HBcAg (**F, G**) were significantly decreased after downregulating or inhibiting NTCP. Scale bar = 200 µm. Results are mean ± SD for three individual experiments. *P < 0.05, **P < 0.01.

To assess whether NTCP downregulation and NTCP blockade interfere with HBV entry and infection in HPCs, we constructed shRNA-NTCP1,2 lentiviral plasmids. We applied real-time quantitative PCR (RT-qPCR) to confirm their ability to reduce NTCP expression in HPCs (Fig. 2A), and shRNA-NTCP#1 was selected at subsequent experiments.

Compared with the control group, the levels of viral components (HBV cccDNA, HBV DNA, HBsAg, HBeAg, and HBcAg) in the HBV group were significantly increased (Fig. 2B through G). The levels of viral components in the HBV + shRNA-NTCP#1 and HBV + shRNA-NTCP#2 groups were significantly decreased compared with those in the HBV group (Fig. 2B through G), whereas the levels of viral components did not differ significantly between the shRNA-NTCP1 and shRNA-NTCP2 groups. These data suggest that podocyte infection with HBV was suppressed after NTCP was downregulated.

### NTCP upregulation enhanced the susceptibility of human podocytes to HBV

**Fig 3:**
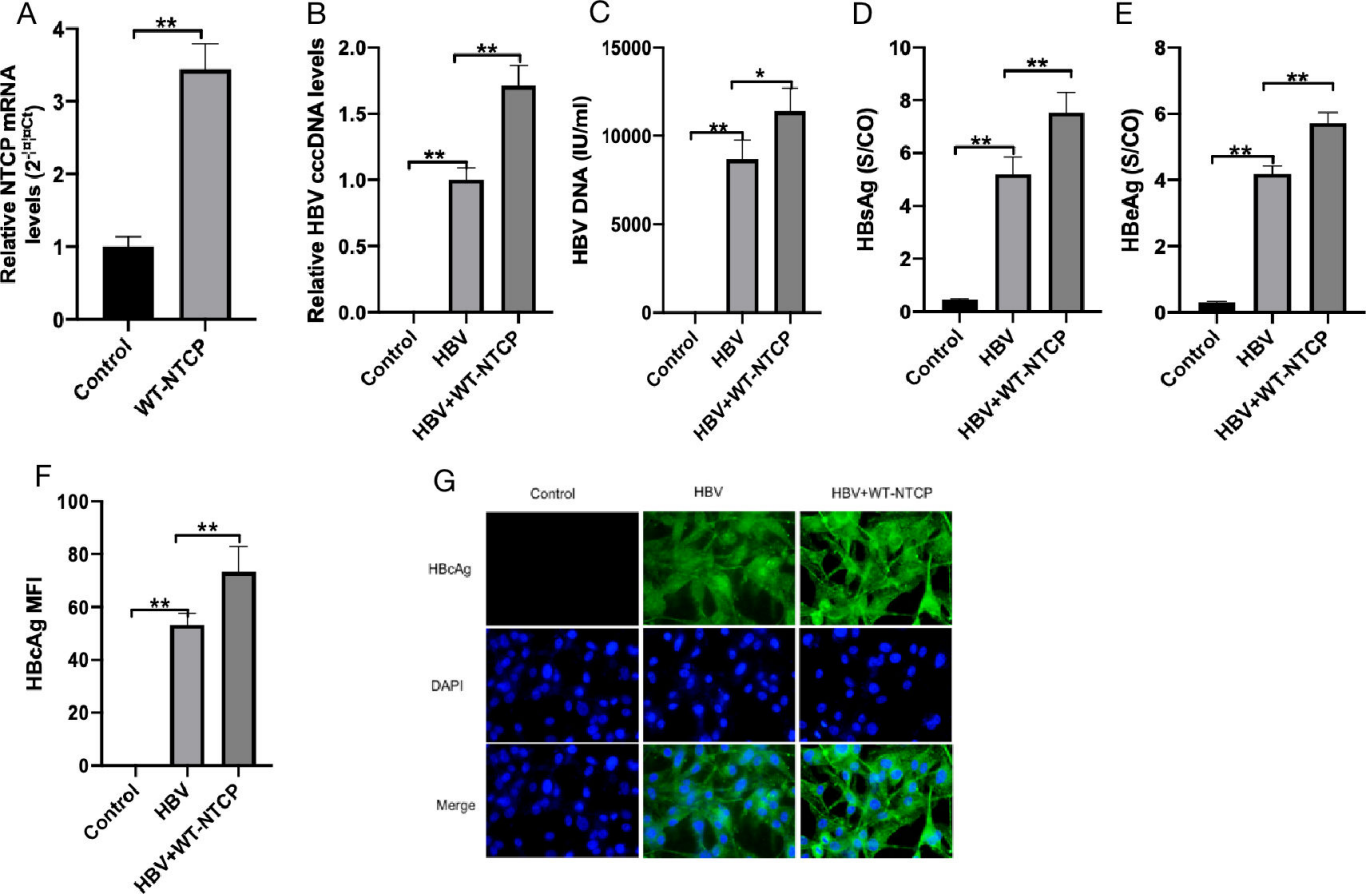
Effect of upregulating NTCP on HBV-infected HPCs. Differentiated podocytes were transfected with WT-NTCP by lentiviral plasimd, and the expression of NTCP measured by qPCR was significantly upregulated after transfection with WT-NTCP (**A**). HPCs in the HBV and HBV + WT-NTCP were incubated with HepG 2.2.15 supernatant (containing HBV) for 16 h, with HPCs incubated with cell culture medium as the control. The levels of HBV cccDNA (**B**) and HBV DNA (**C**) from the indicated groups were measured by RT-qPCR. The levels of HBsAg (**D**) and HBeAg (**E**) in the supernatant from the indicated groups were detected by enzyme-linked immunosorbent assay. (**F, G**) Representative images of HBcAg (green) and 4′,6-diamidino-2-phenylindole nuclear (blue) staining in differentiated HPCs. The levels of HBV cccDNA (**B**), HBV DNA (**C**), HBsAg (**D**), HBeAg (**E**), and HBcAg (**F, G**) were significantly increased after upregulating NTCP. Scale bar = 200 µm. Results are mean ± SD for three individual experiments. *P < 0.05, **P < 0.01.

To further evaluate the effect of NTCP on the HBV susceptibility of HPCs, we constructed WT-NTCP lentiviral plasmids to upregulate NTCP expression and determined the effect of NTCP overexpression on HBV infection. NTCP was upregulated after HPC transfection with WT-NTCP (Fig. 3A). Compared with the control group, the expressions of HBV DNA, HBV cccDNA, HBsAg, HBeAg, and HBcAg in the HBV group were significantly increased (Fig. 3B through G). The levels of HBV cccDNA, HBV DNA, HBsAg, HBeAg, and HBcAg in the HBV + WT-NTCP group were significantly increased compared with those in the HBV group (Fig. 3B through G). These results suggest that the upregulation of NTCP enhances HBV infection in human podocytes.

### Mutation of the NTCP157–165 locus eliminated HBV infection in HPCs

**Fig 4:**
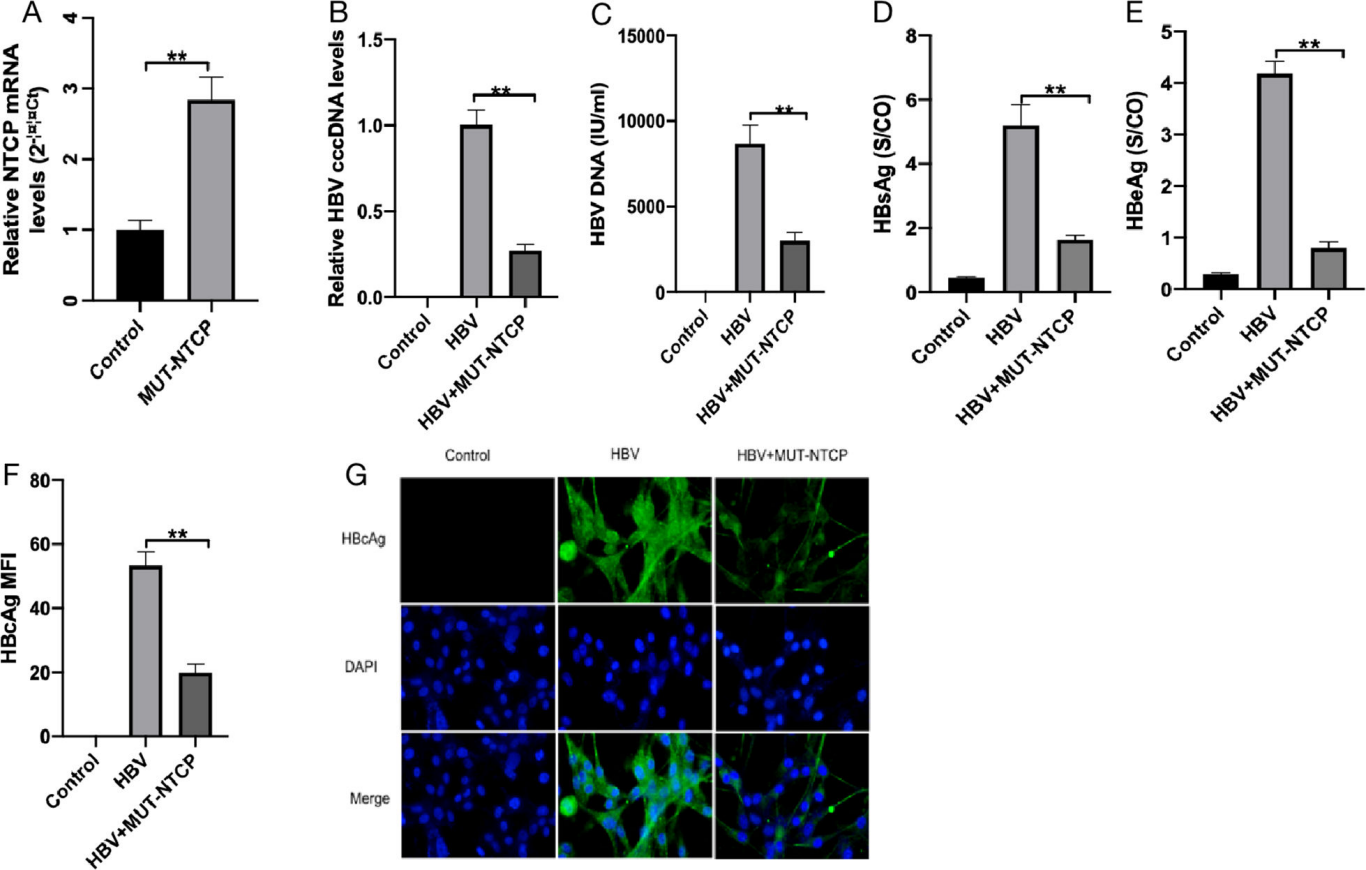
Effect of NTCP mutation on HBV-infected HPCs. Differentiated podocytes were transfected with MUT-NTCP by lentiviral plasmid, and the expression of NTCP was measured by RT-qPCR and was not significantly affected (**A**). HPCs in the HBV and HBV + MUT-NTCP were incubated with HepG 2.2.15 supernatant (containing HBV) for 16 h, with HPCs incubated with cell culture medium as the control. The levels of HBV cccDNA (**B**) and HBV DNA (**C**) from the indicated groups were measured by RT-qPCR. The levels of HBsAg (**D**) and HBeAg (**E**) in the supernatants from the indicated groups were detected by enzyme-linked immunosorbent assay. (**F, G**) Representative images of HBcAg (green) and 4′,6-diamidino-2-phenylindole nuclear (blue) staining in differentiated HPCs. The levels of HBV cccDNA (**B**), HBV DNA (**C**), HBsAg (**D**), HBeAg (**E**), and HBcAg (**F, G**) were significantly decreased after mutating NTCP. Scale bar = 200 µm. Results are mean ± SD for three individual experiments. *P < 0.05, **P < 0.01.

Mutation of the NTCP157–165 locus eliminated the HBV infection of hepatocytes. To further investigate whether NTCP157–165 locus mutation affects the HBV infection of HPCs, we mutated human NTCP157–165 residues to the corresponding residues in monkeys (from KGIVISLVL to GRIILSLVP) and constructed MUT-NTCP lentiviral plasmids to transfect HPCs. The transfection efficiency was determined by RT-qPCR and did not affect the NTCP expression in the HPCs (Fig. 4A). Subsequently, the HPCs were infected with MUT-NTCP plasmid containing HBV supernatant for 5 days. The expressions of HBV DNA, HBV cccDNA, HBsAg, HBeAg, and HBcAg were significantly decreased in the MUT-NTCP group compared with the HBV group (Fig. 4B through G). These data suggest that the NTCP157–165 residues are essential for NTCP-mediated HBV infection in HBV HPCs. The results further support that NTCP is required to mediate HBV infection in HPCs.

### HBV infection downregulates the expression of cytoskeletal protein in HPCs

**Fig 5:**
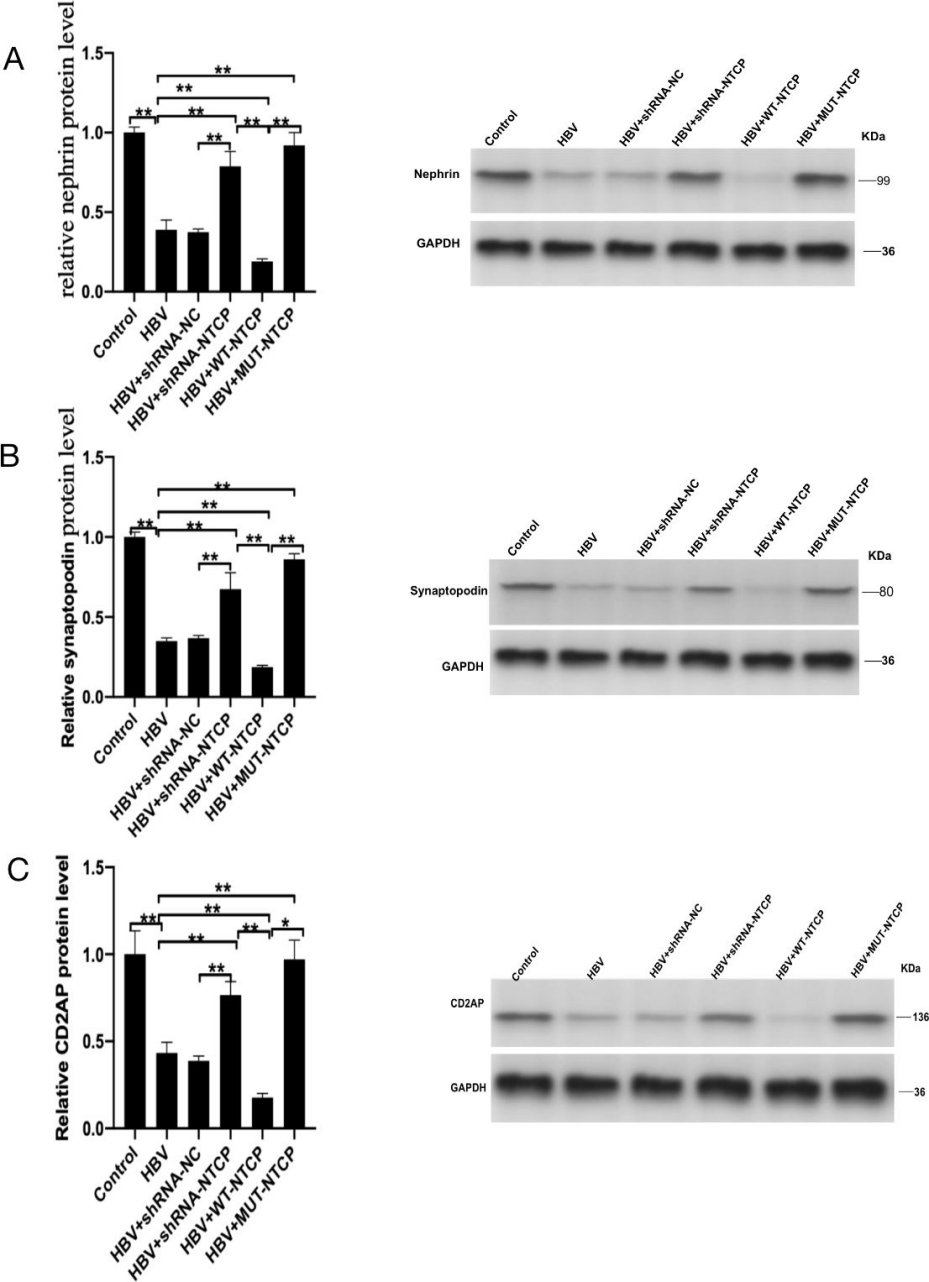
Expression of human podocyte cytoskeletal proteins after HBV infection. Differentiated podocytes were transfected separately with shRNA-NC, shRNA-NTCP, WT-NTCP, and MUT-NTCP by lentiviral plasmid, then incubation with HepG 2.2.15 supernatant for 16 h, and HPCs incubated with cell culture medium as the control. The expressions of nephrin (**A**), synaptopodin (**B**), and CD2AP (**C**) from the indicated groups were detected by western blotting. The levels of nephrin (**A**), synaptopodin (**B**), and CD2AP (**C**) were significantly increased after downregulating, inhibiting, and mutating NTCP. The levels of nephrin (**A**), synaptopodin (**B**), and CD2AP (**C**) were reversed after upregulation of NTCP. In the expression of nephrin (**A**), synaptopodin (**B**), and CD2AP (**C**), GAPDH was repeated as the same internal control experiment. Results are mean ± SD for three individual experiments. *P < 0.05, **P < 0.01.

Podocytes are known to possess unique cytoskeletal structures that are closely related to their physiological function. The expressions of podocyte-specific skeletal proteins—nephrin, synaptopodin, and CD2AP—reflect the degree of podocyte damage. Therefore, we detected the expressions of nephrin, synaptopodin, and CD2AP after HPCs were infected with HBV. The expressions of nephrin, synaptopodin, and CD2AP were significantly decreased in the HBV group compared with those in the control group (Fig. 5A through C). Compared with the HBV group, the expressions of nephrin, synaptopodin, and CD2AP were increased in the HBV + shRNA-NTCP and HBV + MUT-NTCP groups but decreased in the WT-NTCP group. These data indicate that the infection of human podocytes with HBV leads to decreased expressions of human podocyte-specific skeletal proteins, suggesting that podocytes are damaged after HBV infection.

### Apoptosis of HPCs infected with HBV

**Fig 6:**
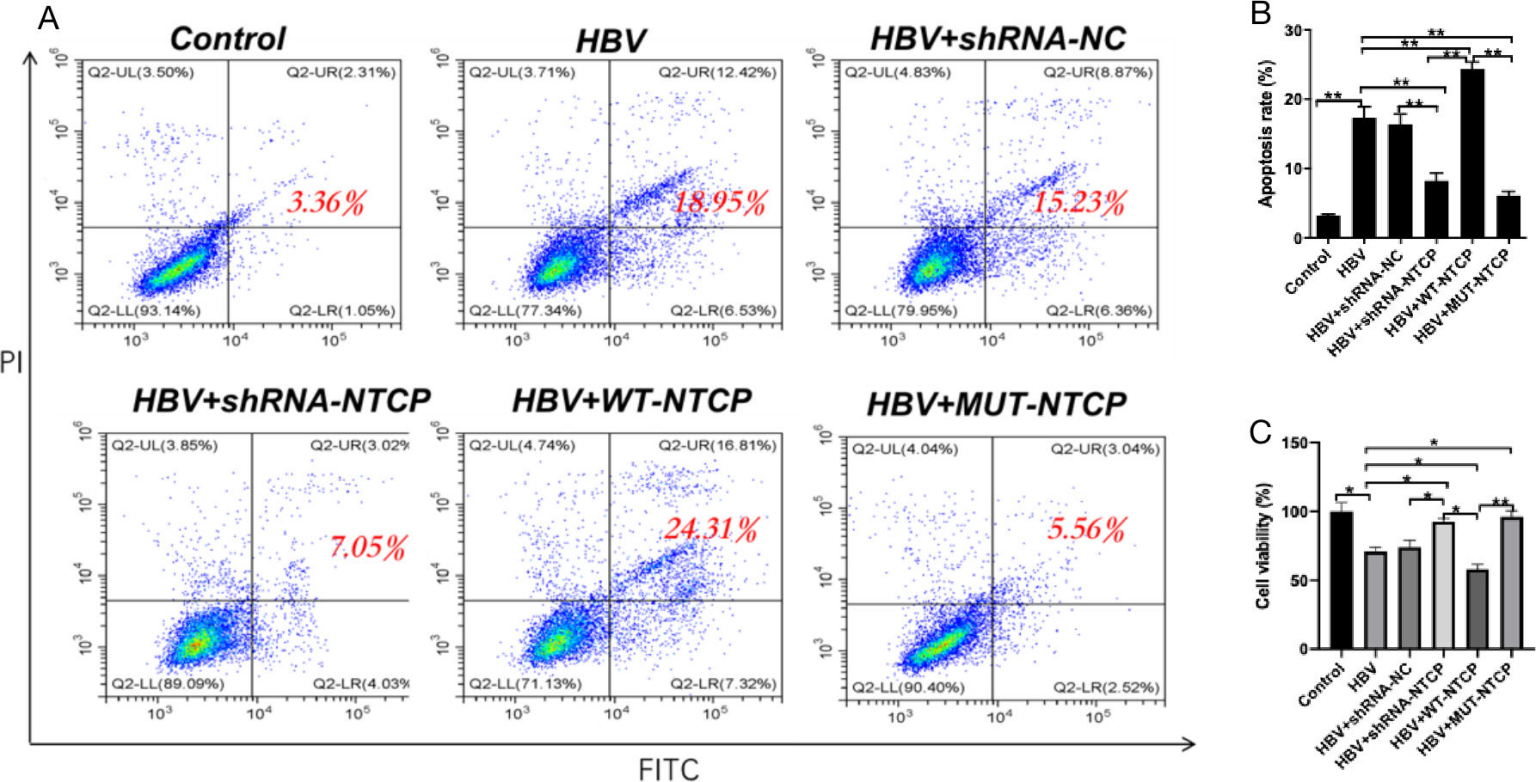
Apoptosis rates of human podocytes after HBV infection. Differentiated podocytes were treated as above. The apoptosis of podocytes (**A, B**) was evaluated by FCM. The cell viability (**C**) of podocytes from the indicated groups was measured by CCK-8 assay. The apoptosis of podocytes (**A, B**) was significantly decreased, and the cell viability (**C**) was significantly increased after downregulating, inhibiting, and mutating NTCP. The apoptosis of podocytes (**A, B**) and the cell viability (**C**) were reversed after upregulation of NTCP. The sum of Q2-UR and Q2-LR is the red number of apoptotic cells. Results are mean ± SD for three individual experiments. *P < 0.05, **P < 0.01.

HPCs were impaired after HBV infection. Podocyte injury can lead to fusion and disappearance of the foot process, eventually resulting in podocyte apoptosis and shedding. Thus, we further assessed whether HBV can induce human podocyte apoptosis. Compared with the control group, the HBV group showed a significant increase in the percentage of apoptotic HPCs and a significant decrease in cell vitality. Compared with the HBV group, the HPCs in the HBV + WT-NTCP group exhibited a higher apoptosis rate and significantly decreased cell vitality. In the HBV + shRNA-NTCP and HBV + MUT-NTCP groups, the apoptosis rate was lower than that in the HBV group, while the cell viability was significantly higher (Fig. 6A through C). These results suggest that the HBV infection of HPCs by HBV exacerbates apoptotic damage in HPCs.

### The proliferation and migration of podocytes were significantly downregulated after HBV infection

**Fig 7:**
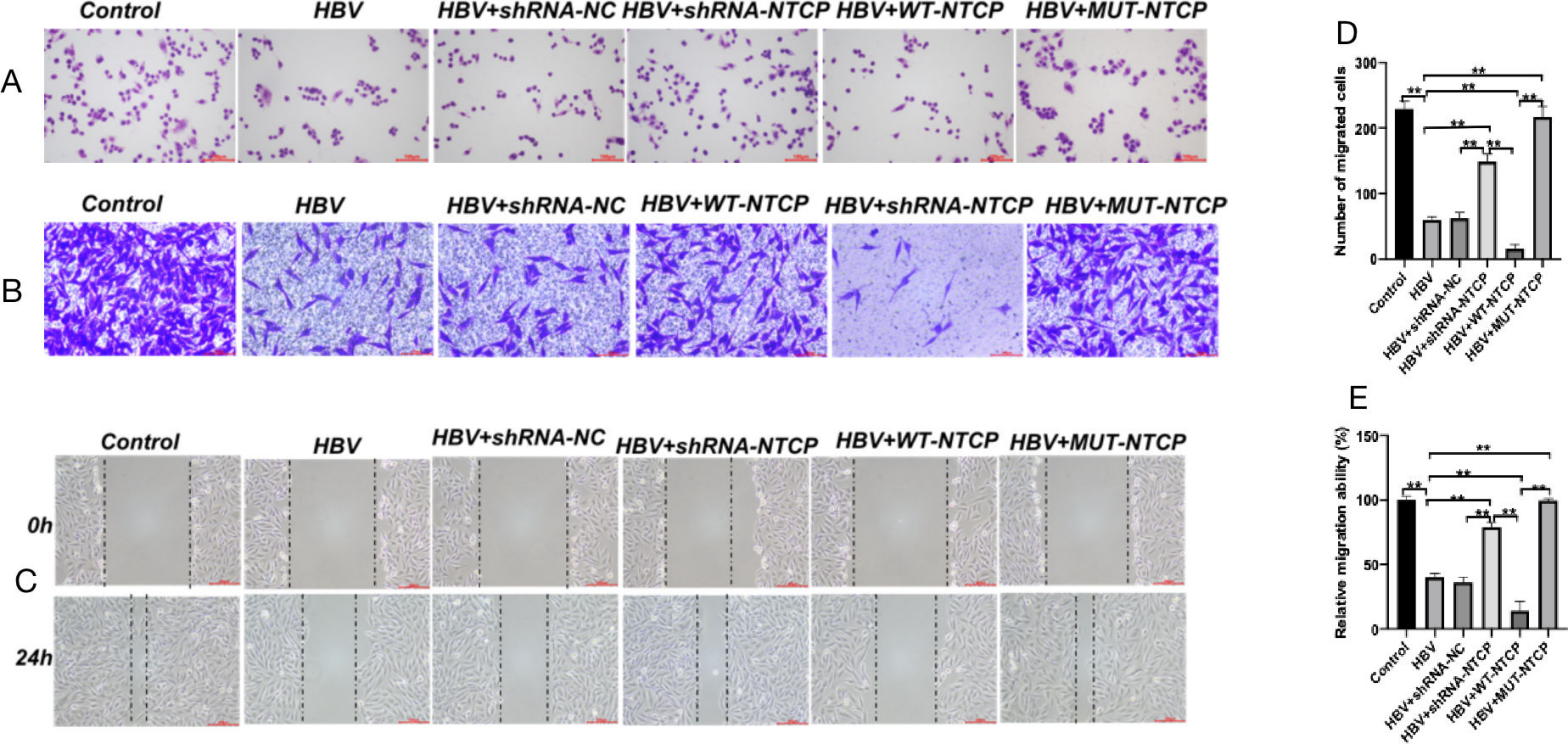
Podocyte proliferation and migration after HBV infection. Differentiated podocytes were treated as above. Cell morphology was evaluated by Wright-Giemsa staining (**A**). Scale bar = 100 µm. Cells that migrated through the Matrigel-free chambers (**B, D**) were stained with crystal violet. Sale bar = 100 µm. Lateral migration was measured by wound healing assay. Representative wound images were captured (**C**) at 0 and 24 h after scratching, and the healed rate was calculated (**E**). Scale bar = 200 µm. Results are mean ± SD for three individual experiments. *P < 0.05, **P < 0.01.

We evaluated the proliferation and migration of human podocytes after HBV infection. Compared with the control group, the cell nuclei of podocytes in the HBV group were heavily stained and shrunken, the cell bodies were smaller, and cell proliferation and migration were decreased significantly (Fig. 7A through E). Compared with the HBV group, the podocytes in the HBV + shRNA-NTCP and HBV + MUT-NTCP groups were normal, and the proliferation and migration of podocytes were enhanced to some extent, whereas the proliferation and migration of podocytes in the HBV + WT-NTCP group were significantly decreased (Fig. 7A through E), and the cell morphology was wrinkled. These results indicate that the repair ability of HPCs is significantly reduced after HBV infection, further aggravating podocyte injury.

## DISCUSSION

HBV-GN is a common extrahepatic disease (17, 18). HBV DNA has been detected in glomerular podocytes and renal tubular epithelial cells (19–22), suggesting that HBV can directly infect renal tissue cells. However, the specific mechanism of the direct HBV infection of renal tissue remains unclear. NTCP is a specific receptor for HBV to enter and infect hepatocytes (16). In fact, in a previous study, we found that NTCP was highly expressed in the glomerular podocytes of HBV-GN patients and human podocytes cultured *in vitro*. Thus, in the present study, we further investigated whether the expression of NTCP in HPCs mediates the HBV infection of HPCs.

Our results show that NTCP-expressing HPCs can be infected by HBV. Previous studies focused on whether NTCP mediates the HBV infection of hepatocytes and did not reveal that NTCP is expressed on human podocyte membranes, or that HPCs can be infected by HBV through NTCP (16). We applied HBV-containing supernatants from Hepg2.2.15 cells to infect HPCs cultured *in vitro* until 9 days. HBV DNA, HBsAg, and HBeAg were detected in human podocytes 1 day after infection, and their expressions peaked 5 days after infection. A previous study conducted in HepG2-NTCP cells found that the expressions of HBV infection indicators peaked at 7 days (23), inconsistent with our results. The difference is likely due to the different cell types. Therefore, our study provides further theoretical support for the pathogenesis of HBV-GN: HBV directly infects renal tissue cells.

To further explore the effect of NTCP on HPC susceptibility to HBV, we constructed plasmids with upregulated NTCP (WT-NTCP) and downregulated NTCP (NTCP-ShRNA) and transfected them into HPCs for HBV infection. We confirmed that HBV susceptibility was significantly increased after NTCP upregulation in HPCs, whereas HBV susceptibility was significantly reduced after downregulating NTCP by shRNA-NTCP. These results are consistent with previous changes in susceptibility to HBV infection after the up- and downregulation and blockade of NTCP in hepatocytes (24). The regulation of NTCP directly affected the HBV infection of HPCs, suggesting that NTCP is necessary for mediating HBV infection in human podocytes.

What is the molecular mechanism by which NTCP mediates the HBV infection of HPCs? HBV infection is strictly species specific, and monkeys cannot be infected by HBV (8, 25). We therefore mutated the human NTCP157–165 locus to the corresponding locus in monkeys and found that the mutation eliminated the ability of HBV to infect human podocytes. This suggests that the human NTCP157–165 residues are a functional locus, consistent with previous studies on HBV infection after mutating NTCP in HepG2-NTCP cells (8, 24). However, HBV-GN occurs only in some HBV-infected individuals, and it is unclear whether there are other sequence limitations of NTCP or other as yet unidentified co-factors that contribute to the occurrence of HBV-GN.

The pathological type of HBV-GN often shows membranous nephropathy-like changes that primarily involve glomerular podocytes and often manifest clinically as massive proteinuria (26). Thus, HBV is presumed to infect HPCs and cause podocyte injury. Podocyte injury can lead to foot process effacement, detachment, apoptosis, proteinuria, and glomerulosclerosis (27–30). In this study, the expressions of nephrin, synaptopodin, and CD2AP, the signature proteins of podocytes, were significantly reduced after HBV infection. In addition, the apoptosis of HPCs increased after HBV infection, while the damage repair and migration abilities of HPCs were significantly decreased. The above changes in the structural proteins and functions of human podocytes were positively correlated with HBV susceptibility. These results suggest that the podocytes were significantly damaged after HBV infection. In a previous clinical study, we found that tacrolimus, a calcium-regulated phosphatase inhibitor, was effective in reducing proteinuria in HBV-GN without increasing viral replication (31). This may be due to the fact that tacrolimus inhibits NTCP to protect podocytes (23, 32). The *in vitro* experimental results of the present study suggest that both HPC damage and functional alterations are consistent with the clinical presentation of HBV-GN patients.

In conclusion, our study provides important information to better understand how HBV directly infects renal tissue cells. The findings provide a new and important theoretical basis for the pathogenesis of HBV-GN (i.e., the direct HBV infection of renal tissue cells) and open the door to investigate blocking NTCP as a new target for HBV-GN therapy. Given that HBV infection damages podocytes, the treatment of HBV-GN should consider the treatment of podocyte damage in addition to the control of HBV infection. In addition, since HBV-GN occurs in only some HBV patients, the specific control of the HBV infection of human renal podocytes may depend on multiple mechanisms, including the interactions of multiple cytokines and membrane proteins. Thus, further studies are needed.

## MATERIALS AND METHODS

### Cell culture and treatment

Conditionally immortalized HPCs were cultured as described previously (33). The culture medium was changed three times per week. Typically, full differentiation occurred in 14 days. For the determination of NTCP expression, the cells were synchronized into quiescence by growing the cells in serum-free Dulbecco’s modified Eagle’s medium (DMEM) for 24 h. The differentiated podocytes were infected with HBV at 10^3^ genome equivalents (GEq) per cell by culturing with the supernatant of HepG2.2.15 cells *in vitro* for 16 h. After discarding the free supernatant, culturing was continued until 9 days after infection. The HBV infection markers were detected at 1, 3 ,5, 7, and 9 days after infection. The peak time of HBV infection was determined experimentally.

HepG2.2.15 cells were grown in DMEM (GIBCO, Invitrogen Corporation, NY, USA) containing penicillin (100 U/mL), streptomycin (100 µg/mL) (Sigma, St. Louis, MO, USA), 1% mycoplasma expectorant (Beyotime Biotechnology, Shanghai, China), and 10% FBS (Hyclone, Logan, UT, USA). The culture medium was changed every other day. The expressions of HBV DNA and HBsAg were detected in the cell supernatant.

### Plasmid transfection

NTCP-shRNA1, NTCP-shRNA2, shRNA control, overexpression plasmid, and mutation plasmid (GRIILSLVP) were synthesized by Ribo Bio Co. (Guangzhou, China) (Table S1). They were constructed into a miR30 context and cloned via XhoI/EcoRI into the lentiviral pAPM vector. Transfection was carried out with the shRNAs using Lipofectamine 2000 (Invitrogen, Thermo Fisher Scientiﬁc, Waltham, MA, USA) transfection reagent following the manufacturer’s protocols. After 48 h of incubation with NTCP shRNA or control shRNA, the cells were treated according to the conditions of the different groups.

### Real-time quantitative PCR for HBV DNA and HBV cccDNA detection

HBV DNA was extracted from HPCs cultured *in vitro* using a QIAamp DNA Mini Kit (Da An Gene Co., Ltd., Zhongshan, Guangzhou, China). HBV DNA was detected and quantified by RT-qPCR using two sets of primers (Table S2). An HBV DNA load greater than or equal to 1 × 10^3^ copies/mL was defined as positive.

The cells were collected and centrifuged at 800 rpm for 5 min. The precipitate was then collected, and 1 mL of lysate without proteinase K was added to every 1 × 10^6^ cells. After incubation at 37°C for 60 min, 0.25 mL of 2.5 M KCl was added to the lysate product followed by shaking, mixing, and centrifugation at 12,000 rpm for 5 min. After this step, all cccDNA and a small amount of rcDNA were located in the supernatant. HBV cccDNA was quantified by real-time PCR using an ABI 7500 Sequence Detection System. The primer sequences are listed in Table S3.

### Detection of HBsAg and HBeAg by enzyme-linked immunosorbent assay

HBsAg and HBeAg were detected in 50 µL volumes of supernatant using commercial enzyme-linked immunosorbent assay (ELISA) kits (Kehua, Shanghai, China) following the manufacturer’s instructions. The ELISA results are presented as the sample-to-cutoff ratio.

### Immunoﬂuorescence detection of HBcAg

The podocytes in each group were inoculated in six-well plates. When the cells proliferated to 70%, the cells were fixed with 500 µL of 4% paraformaldehyde for 15 min. After washing three times with phosphate-buffered saline (PBS) and permeabilizing with 0.5% Triton X-100, primary antibody against HBcAg (ab8639 Abcam, Cambridge, 1:100) was added and incubated overnight at 4°C. Subsequently, the cells were incubated with secondary antibody (goat anti-mouse IgG H&L; Alexa Fluor 488; ab150113 Abcam, Cambridge, 1:1,000), washed three times with PBS, sealed with 4′,6-diamidino-2-phenylindole, and observed under a laser confocal microscope (Zeiss LSM, Zeiss, Germany). The field of view for imaging was randomly selected.

### Western blotting

Western blotting was performed as described previously (34). An aliquot of cell lysates containing 40 mg of protein was separated on 10% sodium dodecyl sulfate–polyacrylamide gels and then transferred to polyvinylidene fluoride membranes by electroblotting. The electroblotted membranes were immersed in a blocking solution that contained 5% nonfat dry milk and TBS-T [0.05% Tween 20, 20 mmol/L Tris-HCl, and 150 mmol/L NaCl (pH 7.6)]. Membranes were then incubated overnight at 4°C with the following primary antibodies and secondary antibodies: NTCP rabbit polyclonal antibody (2 µg/mL; GTX 17693, Gene Tex, USA), anti-GAPDH antibody (ab8245, Abcam, Cambridge, 1:1,000), and goat anti-rabbit IgG H&L (HRP) (ab6721, Abcam, Cambridge, 1:10,000). CD2AP (2135, Cell Signaling Technology, Boston, USA, 1:1,000); Nephrin (ab235903, Abcam, Cambridge, 1:1,000); Synaptopodin (ab224491, Abcam, Cambridge, 0.4 µg/mL). Image Pro Plus 6.0 software was used to analyze the optical density value, and the relative protein expression was calculated as the target protein gray value/internal reference protein gray value.

### Detection of podocyte apoptosis by flow cytometry

Apoptotic cells in different groups were determined using an Annexin V/PI apoptosis detection kit according to the manufacturer’s protocol (Nanjing KeyGEN Biotech, China). Cell fluorescence was then analyzed using a Beckman DxFlex (Beckman Colter, Inc., USA). Cells positive for Annexin V-FITC were considered apoptotic. Apoptotic cells were expressed as the percentage of the total cells.

### Detection of podocyte migration by wound healing assay

Wound healing assay was performed as described previously (35, 36). Podocytes were grown in six-well dishes overnight and then treated with and without HBV. Each cell was scratched with a sterile 200-µL pipette tip and washed with PBS. The number of migrated cells was estimated using phase-contrast microscopy (Nikon Olympus, Tokyo, Japan) at 0 and 24 h after scratching. Wound closure was measured using ImageJ software (National Institutes of Health, Bethesda, MD, USA).

### Transwell migration assay

For each experiment, 2.5 × 10^5^ cultured differentiated HPCs per milliliter were seeded in Transwell cell culture inserts (BD Corporation, USA) and allowed to migrate for 24 h while being incubated at 37°C. Non-migratory cells were removed from the upper surface of the membrane, and migrated cells were fixed with 4% paraformaldehyde and stained with crystal violet solution (Sigma-Aldrich, USA). The number of migrated cells was counted using phase-contrast microscopy with a 20× objective (Nikon Olympus, Tokyo, Japan) in the center of a membrane (one field). The data presented represent the mean of three independent experiments.

### Wright-Giemsa staining

After treating the cells according to the experimental groups, the cell slides were taken out, fixed with 70% ethanol solution for 10 min, and then stained with the configured Wright-Giemsa working solution (1×) for 45 min. The slides were then washed with distilled water from one side. After drying, the slides were observed under a microscope, and images were captured.

### Statistical analysis

Results are expressed as the mean ± standard deviation (SD). Statistical analysis was performed using one-way analysis of variance (ANOVA) with Turkey’s *post hoc* test (GraphPad Prism8.0, GraphPad Software, Inc., La Jolla, CA, USA). *P* < 0.05 was considered to indicate a significant difference.

## Data Availability

The data that support the findings of this study can be found in the supplemental material.

## SUPPLEMENTAL MATERIAL

Tables S1-S3 spectrum.01365-23-s0001.docx Primers used in this study.

Supplemental data spectrum.01365-23-s0002.pdf Data for Fig. 1 to 7.

ASM does not own the copyrights to Supplemental Material that may be linked to, or accessed through, an article. The authors have granted ASM a non-exclusive, world-wide license to publish the Supplemental Material files. Please contact the corresponding author directly for reuse.
