# Supplementary material for: Hepatitis B virus-targeting sodium taurocholate cotransporting polypeptide mediates HBV infection and damage in human renal podocytes
Source: Microbiol Spectr. 2024 Feb 5;12(3):e01365-23. doi: 10.1128/spectrum.01365-23 (PMC10913464; doi:10.1128/spectrum.01365-23)
Supplement: Tables S1-S3 — Primers used in this study. [file spectrum.01365-23-s0001.docx]

Supplementary

Table S1: Primers for NTCP-shRNA1, NTCP-shRNA2, NTCP-shRNA control

and WT-NTCP used in this study

|  | Sequence |
| --- | --- |
| NTCP-shRNA1 | 5-TGCACCATGGAGTTCAGCA |
| NTCP-shRNA2 | 5-ATGGAGTTCAGCAAGATCA |
| NTCP-shRNA control | 5-CTCGCTTG GGCGAG AGTAA |
| WT-NTCP | F:GTTTGGATCCATGGAGG CCCACAA CGCGTCTGCCC R:CGCCACTAGTCTAGGCTGTGCAAGGGGAGCAGTCCTC |

Table S2: Primers for HBV DNA used in this study

|  | sequences |
| --- | --- |
| HBV DNA | 5’-ATCCTGCTGCTATGCCT CAT CTT-3’ (forward, F)  5’-ACAGTGGGGGAAAGCCCTACGAA-3’ (reverse, R) |

Table S3: Primers for HBV DNA used in this study

|  | sequences |
| --- | --- |
| HBV cccDNA | 5’-TGCACTTCGCTTCACCT-3’ (forward, F)  5’-AGGGGCATTTGGTGGTC-3’ (reverse, R) |
