# Supplementary material for: Hepatitis B virus-targeting sodium taurocholate cotransporting polypeptide mediates HBV infection and damage in human renal podocytes
Source: Microbiol Spectr. 2024 Feb 5;12(3):e01365-23. doi: 10.1128/spectrum.01365-23 (PMC10913464; doi:10.1128/spectrum.01365-23)
Supplement: Supplemental data — Data for Fig. 1 to 7. [file spectrum.01365-23-s0002.pdf]

论文原始数据

FIGURE1

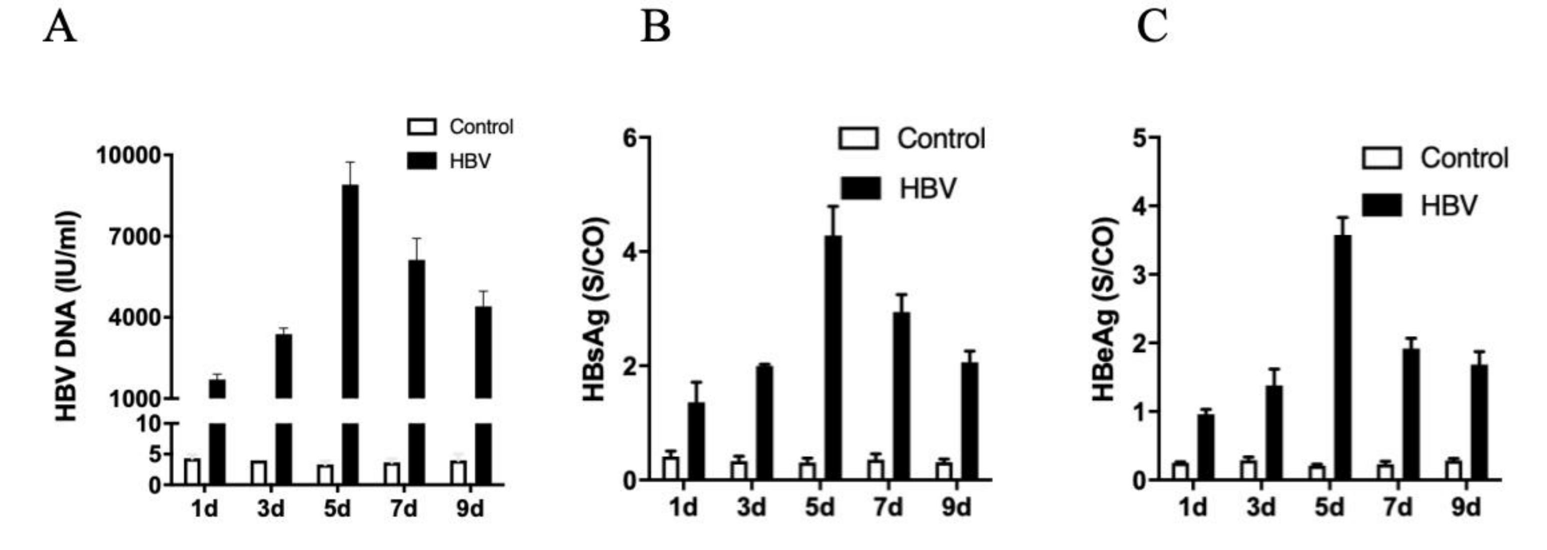
A
B
C
Control
HBV
HBV DNA (IU/ml)
10000
7000
4000
1000
10
5
0
1d
3d
5d
7d
9d
HBsAg (S/CO)
6
4
2
0
HBeAg (S/CO)
5
4
3
2
1
0

FIGURE1A

	HepG2.2.15
CT	17.71
	17.81
	18.17
Average	17.8967
SD	0.24194

	Potocytes					Potocytes+HBV				
	1d	3d	5d	7d	9d	1d	3d	5d	7d	9d
CT	33.51	33.4	33.47	33.3	33.62	24.31	23.05	21.68	22.15	22.8
	33.49	33.66	33.82	33.35	33.23	23.96	23.13	21.54	21.91	22.42
	33.23	33.53	33.8	33.7	33.81	24.04	22.93	21.39	22.3	22.67
Average	33.41	33.53	33.6967	33.45	33.5533	24.1033	23.0367	21.5367	22.12	22.63
SD	0.1562	0.13	0.19655	0.21794	0.29569	0.18339	0.10066	0.14503	0.19672	0.19313

	HepG2.2.15
HBV DNA (IU/ml)	104822
	98273
	77905
Average	93666.7
SD	14037.3

	Potocytes					Potocytes+HBV				
	1d	3d	5d	7d	9d	1d	3d	5d	7d	9d
/ DNA (IU	4	4	4	4	4	1483	3344	8093	5976	3929
	4	4	3	4	5	1859	3176	8858	6977	5021
	5	4	3	3	3	1765	3613	9758	5425	4273
Average	4.33333	4	3.33333	3.66667	4	1702.33	3377.67	8903	6126	4407.67
SD	0.57735	0	0.57735	0.57735	1	195.677	220.437	833.412	786.798	558.317

FIGURE1B

	Group A			Group B		
	Potocytes			Potocytes+HBV		
	A:Y1	A:Y2	A:Y3	B:Y1	B:Y2	B:Y3
1d	0.41	0.51	0.32	1.27	1.75	1.06
3d	0.24	0.35	0.41	1.97	2.03	1.99
5d	0.36	0.22	0.34	3.75	4.33	4.77
7d	0.42	0.24	0.41	3.27	2.66	2.89
9d	0.25	0.32	0.36	1.84	2.15	2.20
[illegible]						

HIGURE1C

	Potocytes			Potocytes+HBV		
	A:Y1	A:Y2	A:Y3	B:Y1	B:Y2	B:Y3
1d	0.26	0.24	0.26	1.02	0.88	0.98
3d	0.34	0.28	0.25	1.20	1.65	1.29
5d	0.19	0.21	0.23	3.67	3.29	3.77
7d	0.20	0.28	0.21	1.99	2.02	1.74
9d	0.29	0.31	0.25	1.69	1.87	1.49

figure2A/B/C

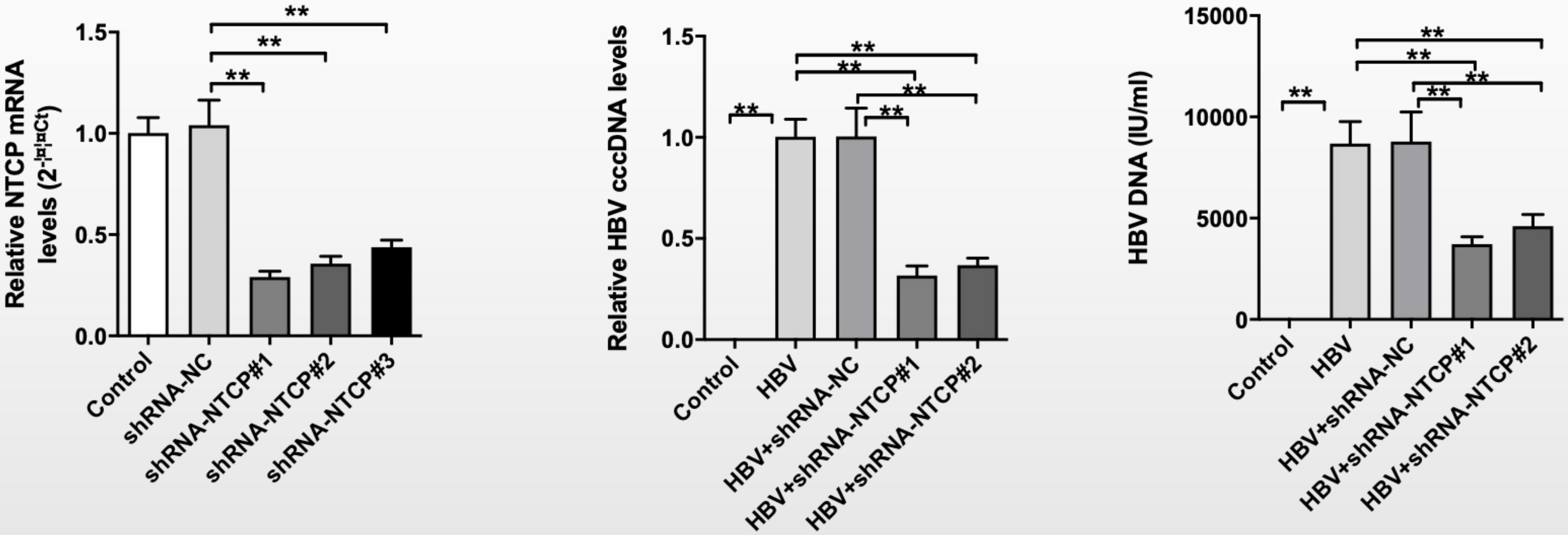
Relative NTCP mRNA levels
1.5
1.0
0.5
0.0
**
Control
shRNA-NC
shRNA-NTCP#1
shRNA-NTCP#2
shRNA-NTCP#3
Relative HBV cccDNA levels
Control
HBV
HBV+shRNA-NC
HBV+shRNA-NTCP#1
HBV+shRNA-NTCP#2
HBV DNA (IU/ml)
15000
10000
5000
0

FIGURE2A/B/C

2A

Control	shRNA-NC	shRNA-NTCP#1	shRNA-NTCP#2	shRNA-NTCP#3
Y	Y	Y	Y	Y
0.9954	1.1045	0.2839	0.3261	0.4676
0.9287	0.8971	0.3216	0.3447	0.3987
1.0817	1.1199	0.2667	0.3959	0.4454

2B

	Control	HBV	HBV+shRNA-NC	HBV+shRNA-NTCP#1	HBV+shRNA-NTCP#2
	Y	Y	Y	Y	Y
1	0.0005	1.0918	1.0619	0.2618	0.3479
2	0.0007	0.9181	1.1070	0.3407	0.4080
3	0.0008	0.9977	0.8448	0.3479	0.3479

2C

Control	HBV	HBV+shRNA-NC	HBV+shRNA-NTCP#1	HBV+shRNA-NTCP#2
Y	Y	Y	Y	Y
9.00	8526.00	7346.00	3720.00	5210.00
12.00	9833.00	8694.00	3353.00	4547.00
10.00	7687.00	10289.00	4073.00	4047.00

figure2D/E/F

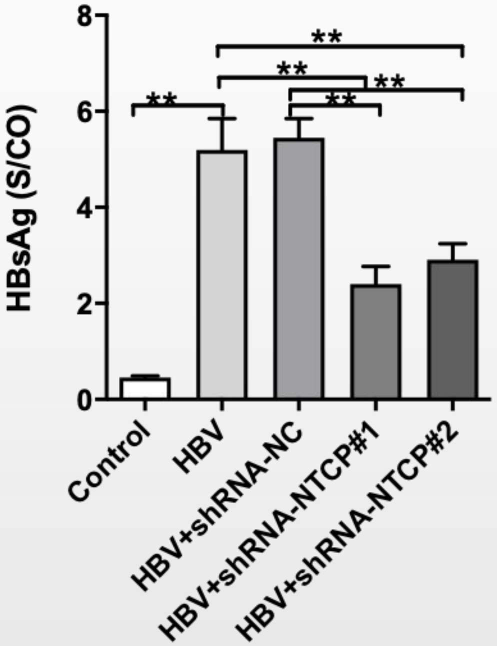

HBsAg (S/CO)
8
6
4
2
0
**
**
**
**
**
Control
HBV
HBV+shRNA-NC
HBV+shRNA-NTCP#1
HBV+shRNA-NTCP#2

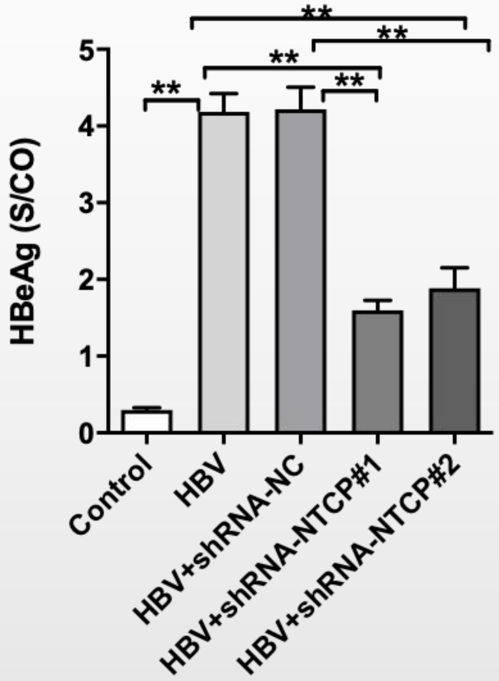

HBeAg (S/CO)
5
4
3
2
1
0
**
**
**
**
**
Control
HBV
HBV+shRNA-NC
HBV+shRNA-NTCP#1
HBV+shRNA-NTCP#2

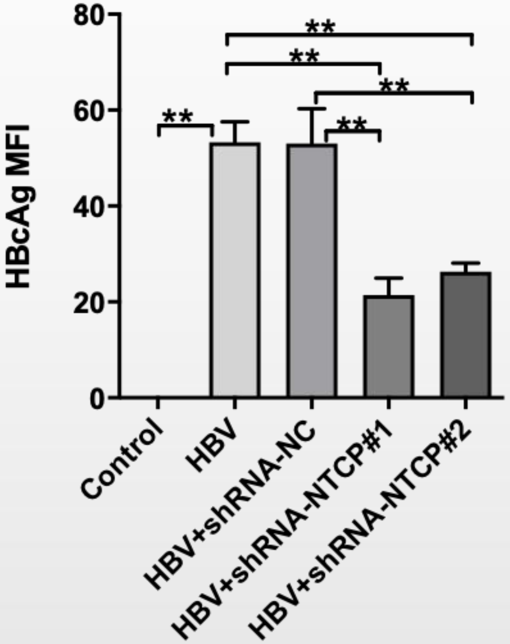

HBcAg MFI
80
60
40
20
0
**
**
**
**
**
Control
HBV
HBV+shRNA-NC
HBV+shRNA-NTCP#1
HBV+shRNA-NTCP#2

figure2D/E/F

2D

Control	HBV	HBV+shRNA-NC	HBV+shRNA-NTCP#1	HBV+shRNA-NTCP#2
Y	Y	Y	Y	Y
0.45	4.47	5.90	2.38	2.84
0.43	5.38	5.14	2.05	2.62
0.49	5.74	5.31	2.78	3.27

2E

Control	HBV	HBV+shRNA-NC	HBV+shRNA-NTCP#1	HBV+shRNA-NTCP#2
Y	Y	Y	Y	Y
0.32	3.93	4.32	1.45	1.82
0.26	4.24	4.44	1.64	2.18
0.31	4.39	3.89	1.70	1.65

2F

Control	HBV	HBV+shRNA-NC	HBV+shRNA-NTCP#1	HBV+shRNA-NTCP#2
Y	Y	Y	Y	Y
0.0000	53.3000	54.7500	25.5100	26.8300
0.0000	57.5600	45.1000	19.7900	27.7700
0.0000	49.0300	59.3100	18.9200	24.3800

figure2G

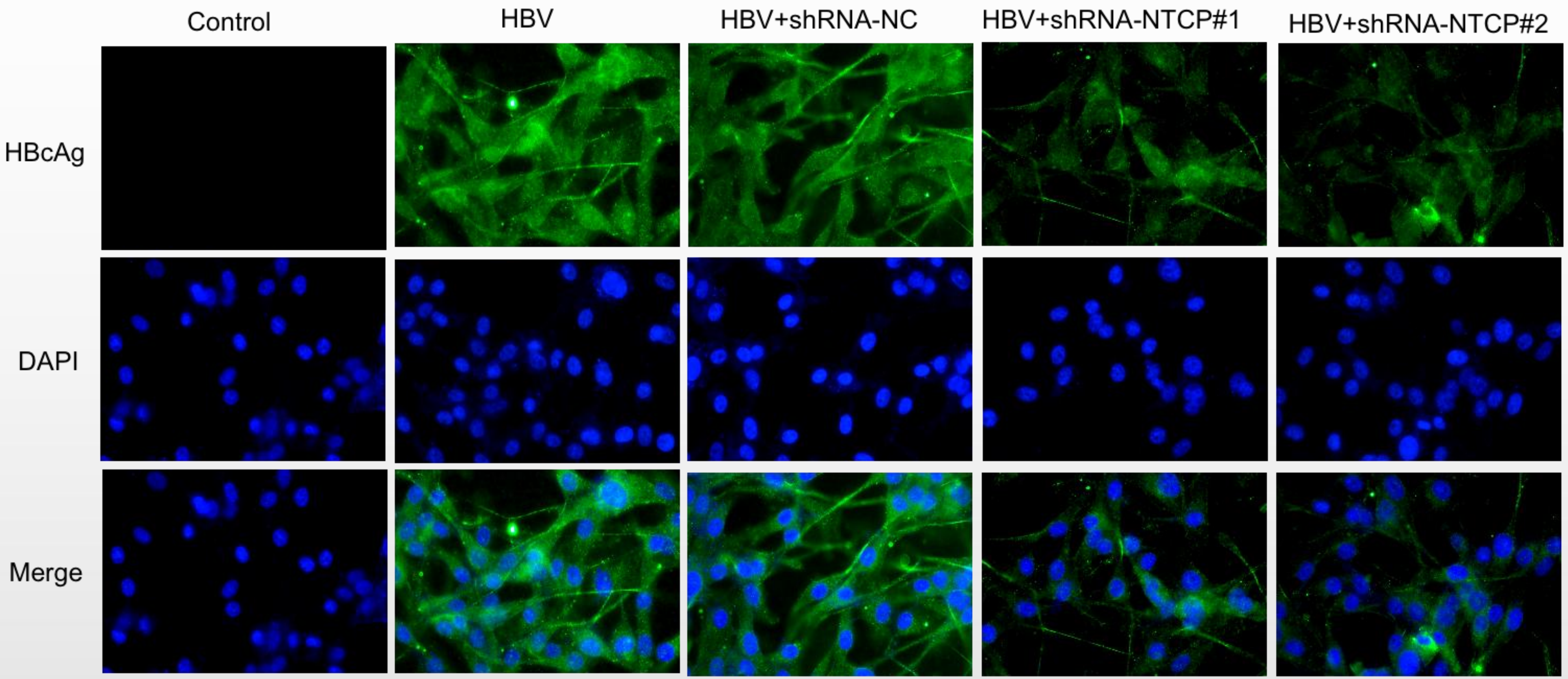

Control
HBV
HBV+shRNA-NC
HBV+shRNA-NTCP#1
HBV+shRNA-NTCP#2
HBcAg
DAPI
Merge

figure3A/B/C/D/E/F

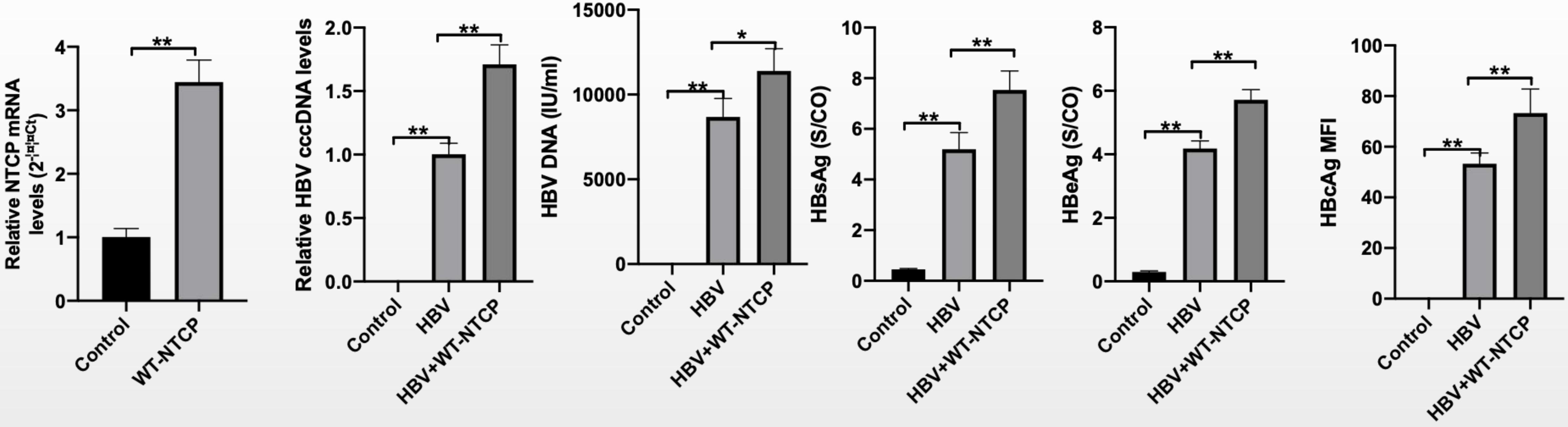

figure3A/B/C/D/E/F

NTCP mRNA			HBV cccDNA				HBV DNA		
Control	WT-NTCP		Control	HBV	WT-NTCP		Control	HBV	WT-NTCP
1.0619	3.4263		0.0005	1.0918	1.8489		9	8526	10628
0.8566	3.1095		0.0007	0.9181	1.7371		12	9833	12909
1.0994	3.8018		0.0008	0.9977	1.544		10	7687	10628
HBsAg			HBeAg				HBcAg		
Control	HBV	WT-NTC	Control	HBV	V+WT-NTCP		Control	HBV	V+WT-NTCP
0.45	4.47	8.29	0.32	3.93	6.07		0	53.3	79.57
0.43	5.38	6.79	0.26	4.24	5.63		0	57.56	77.94
0.49	5.74	7.54	0.31	4.39	5.44		0	49.03	62.42

FIGURE3G

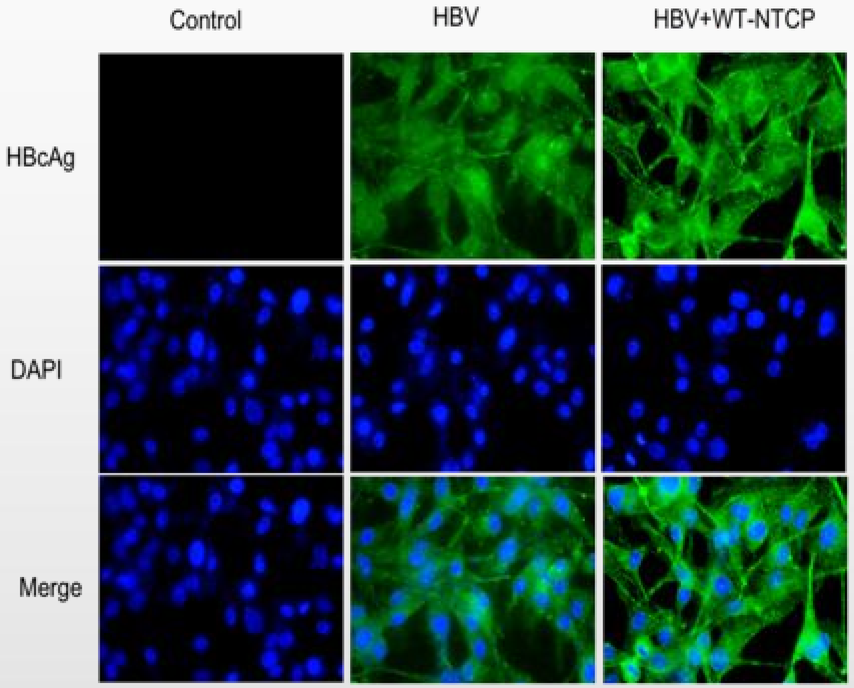

Figure4A/B/C/D/E/F

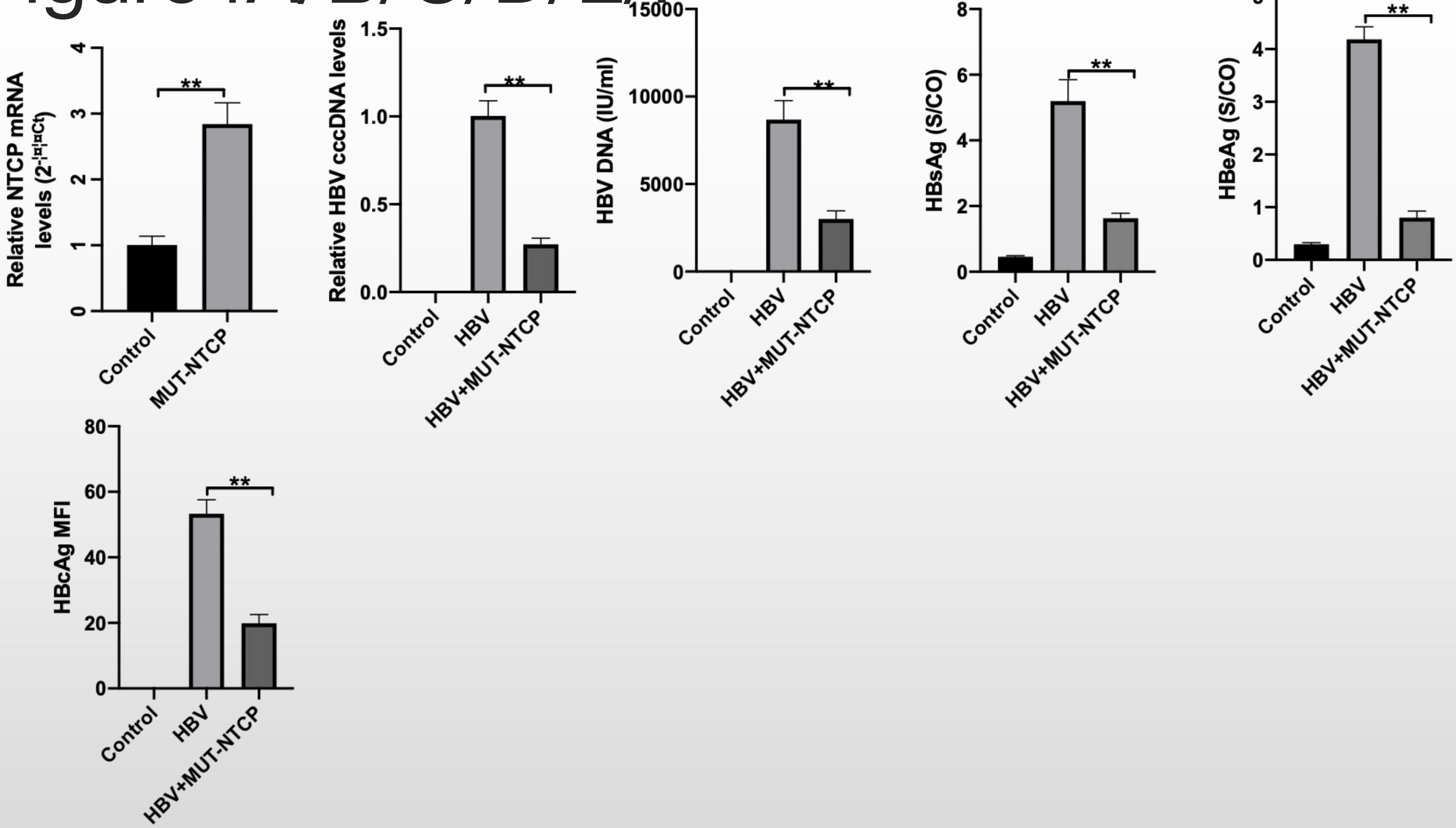

Figure4A/B/C/D/E/F

NTCP mRNA	
Control	MUT-NTCP
1.0619	2.8219
0.8566	2.5257
1.0994	3.1748

HBV cccDNA		
Control	HBV	/+MUT-NTCP
0.0005	1.0918	0.2426
0.0007	0.9181	0.3114
0.0008	0.9977	0.2618

HBV DNA		
Control	HBV	/+MUT-NTCP
9	8526	2473
12	9833	3310
10	7687	3225

HBsAg		
Control	HBV	/+MUT-N
0.45	4.47	1.61
0.43	5.38	1.79
0.49	5.74	1.48

HBeAg		
Control	HBV	/+MUT-NTCP
0.32	3.93	0.8
0.26	4.24	0.68
0.31	4.39	0.93

HBcAg		
Control	HBV	/+MUT-NTCP
0	53.3	20.1
0	57.56	17.13
0	49.03	22.42

FIGURE4G

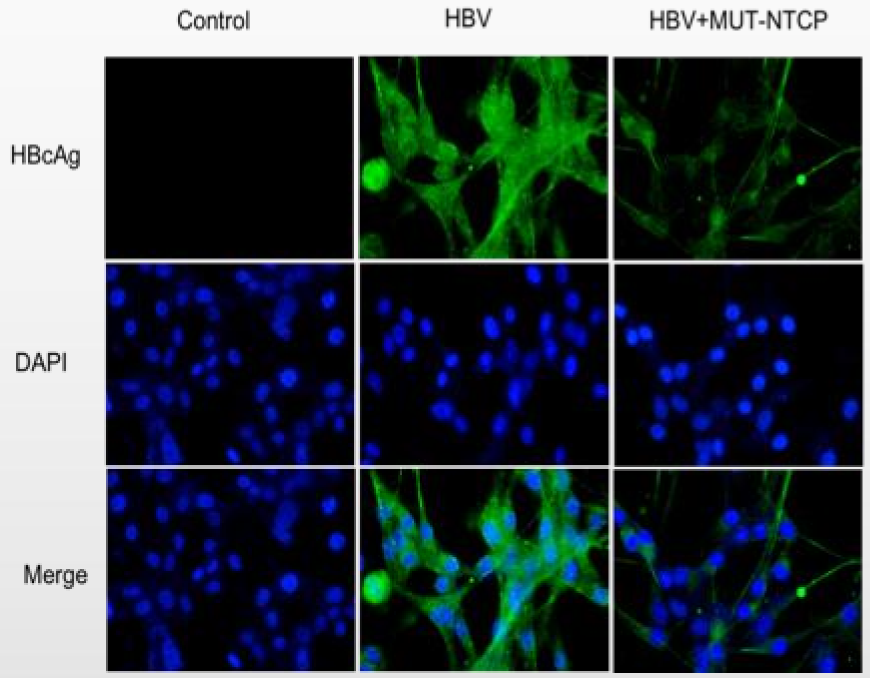

FIGURE5A

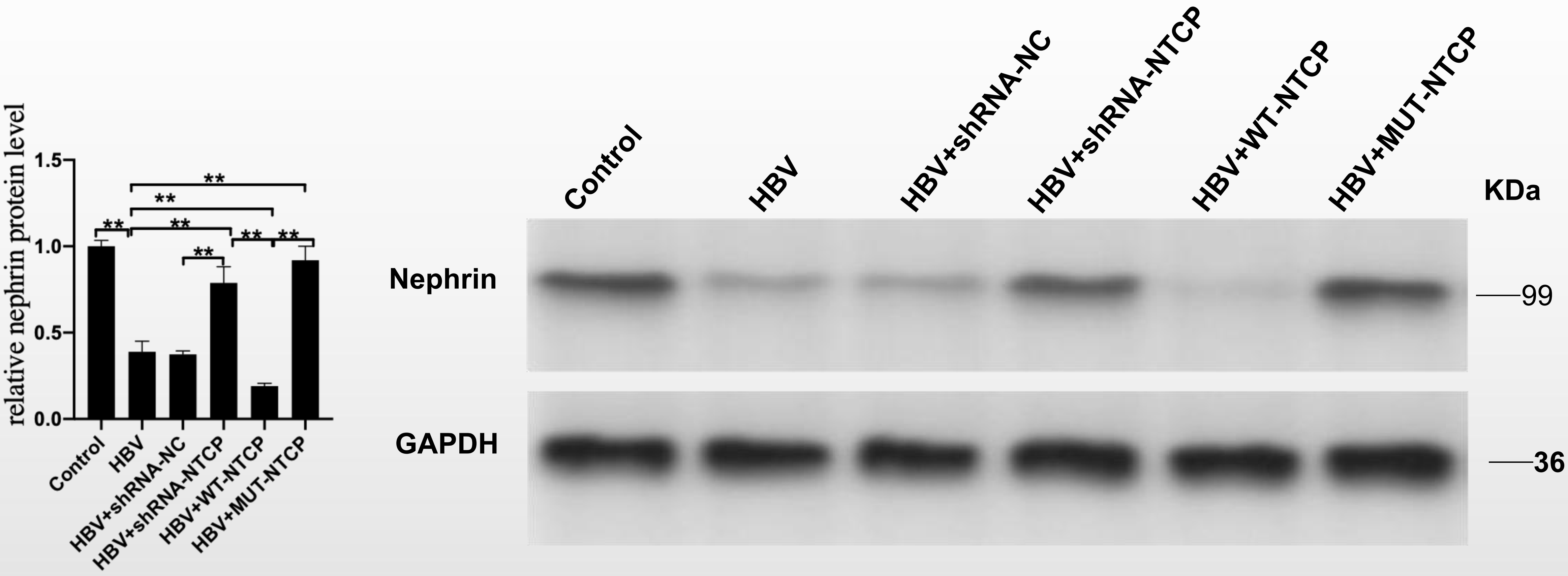
relative nephrin protein level
1.5
1.0
0.5
0.0
**
Control
HBV
HBV+shRNA-NC
HBV+shRNA-NTCP
HBV+WT-NTCP
HBV+MUT-NTCP
KDa
Nephrin
GAPDH
99
36

FIGURE5A

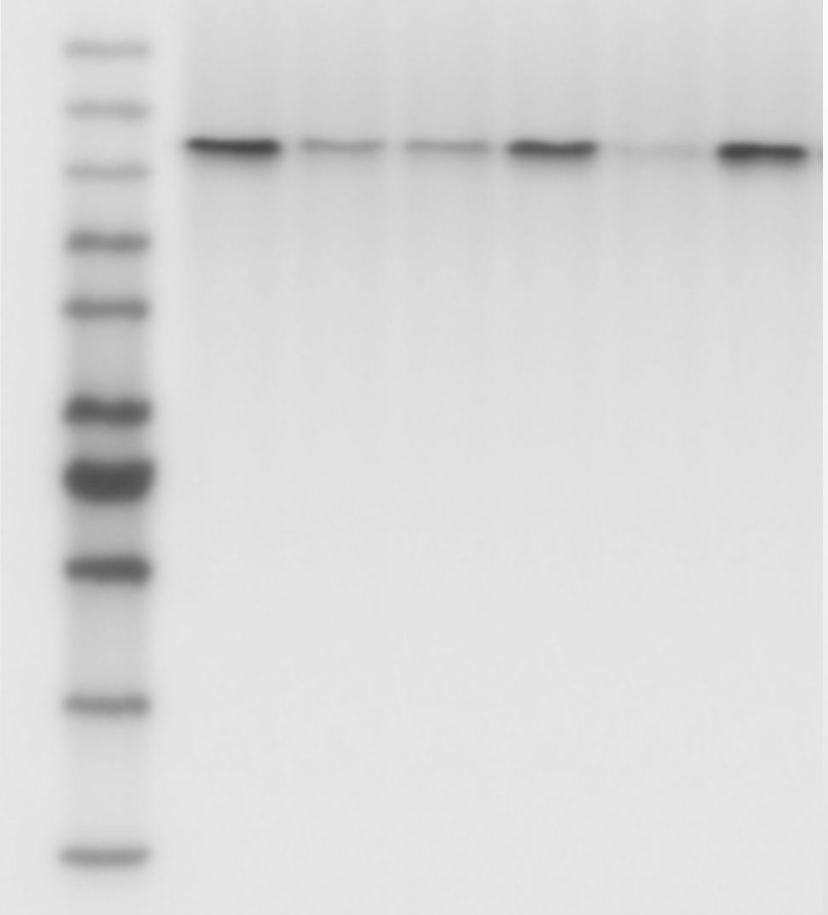

Control	HBV	HBV+shRNA-NC	HBV+shRNA-NTCP	HBV+WT-NTCP	HBV+MUT-NTCP
Y	Y	Y	Y	Y	Y
1.0001	0.3476	0.3596	0.8206	0.1743	0.9537
0.9651	0.3625	0.3654	0.6804	0.2075	0.8269
1.0349	0.4592	0.3967	0.8597	0.1900	0.9780

FIGURE5B

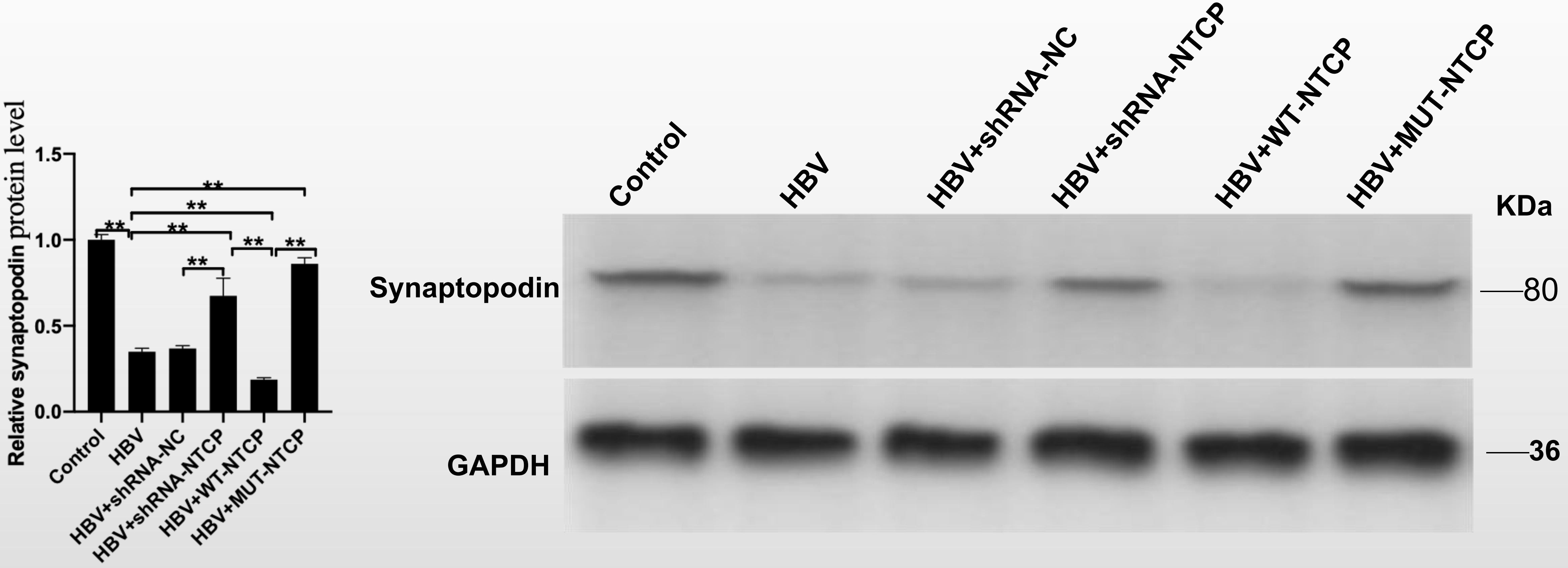

Relative synaptopodin protein level
1.5
1.0
0.5
0.0
**
Control
HBV
HBV+shRNA-NC
HBV+shRNA-NTCP
HBV+WT-NTCP
HBV+MUT-NTCP
KDa
Synaptopodin
80
GAPDH
36

FIGURE5B

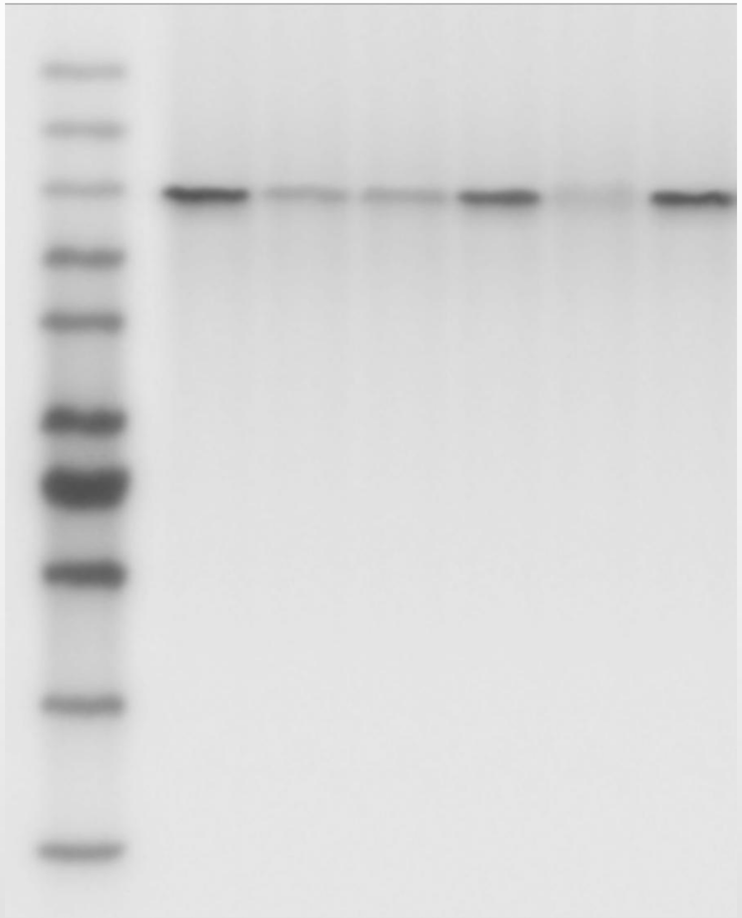

Control	HBV	HBV+shRNA-NC	HBV+shRNA-NTCP	HBV+WT-NTCP	HBV+MUT-NTCP
Y	Y	Y	Y	Y	Y
1.0067	0.3656	0.3766	0.7911	0.1912	0.8883
1.0262	0.3262	0.3496	0.5972	0.1745	0.8212
0.9672	0.3558	0.3784	0.6351	0.1946	0.8716

figure5c

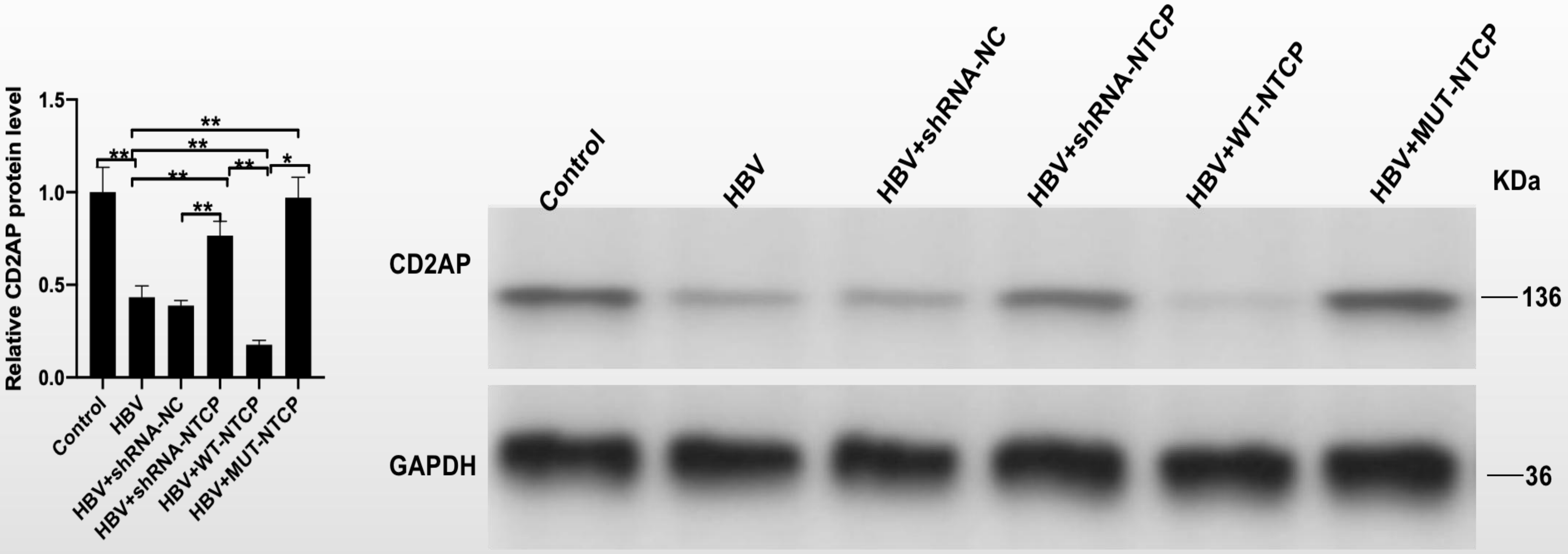

Relative CD2AP protein level
1.5
1.0
0.5
0.0
**
**
**
**
**
**
*
Control
HBV
HBV+shRNA-NC
HBV+shRNA-NTCP
HBV+WT-NTCP
HBV+MUT-NTCP
KDa
CD2AP
GAPDH
136
36

FIGURE5C

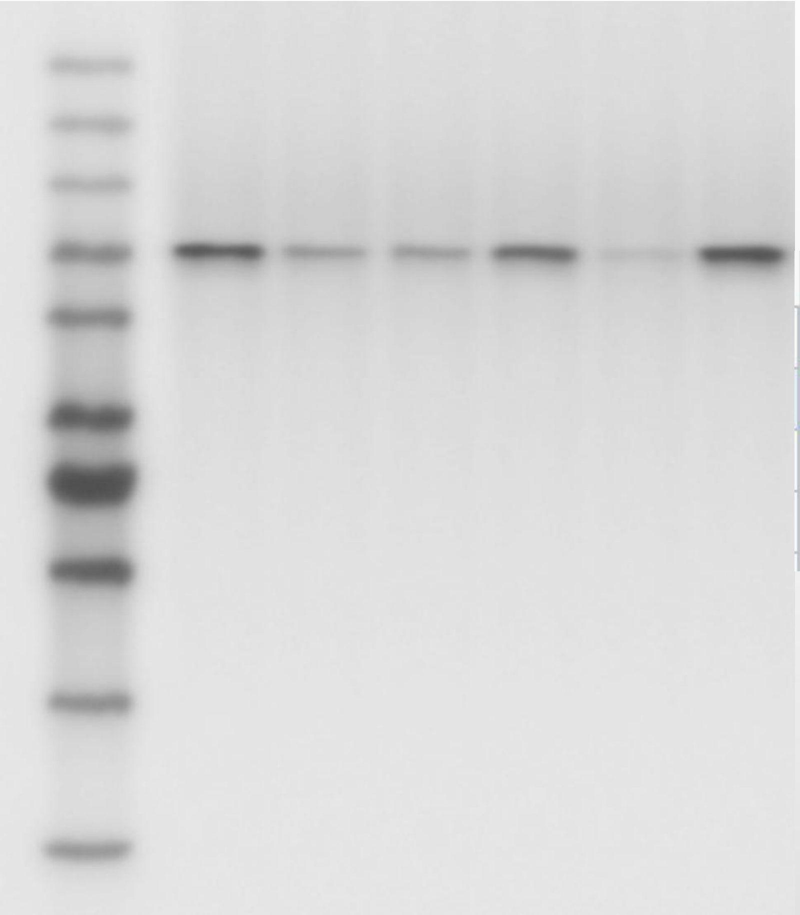

Control	HBV	HBV+shRNA-NC	HBV+shRNA-NTCP	HBV+WT-NTCP	HBV+MUT-NTCP
Y	Y	Y	Y	Y	Y
0.9201	0.3695	0.3686	0.7102	0.1593	0.9149
0.9248	0.4352	0.3756	0.7303	0.1671	0.8982
1.1551	0.4936	0.4194	0.8545	0.2042	1.0978

FIGURE5A/B/C: GAPDH

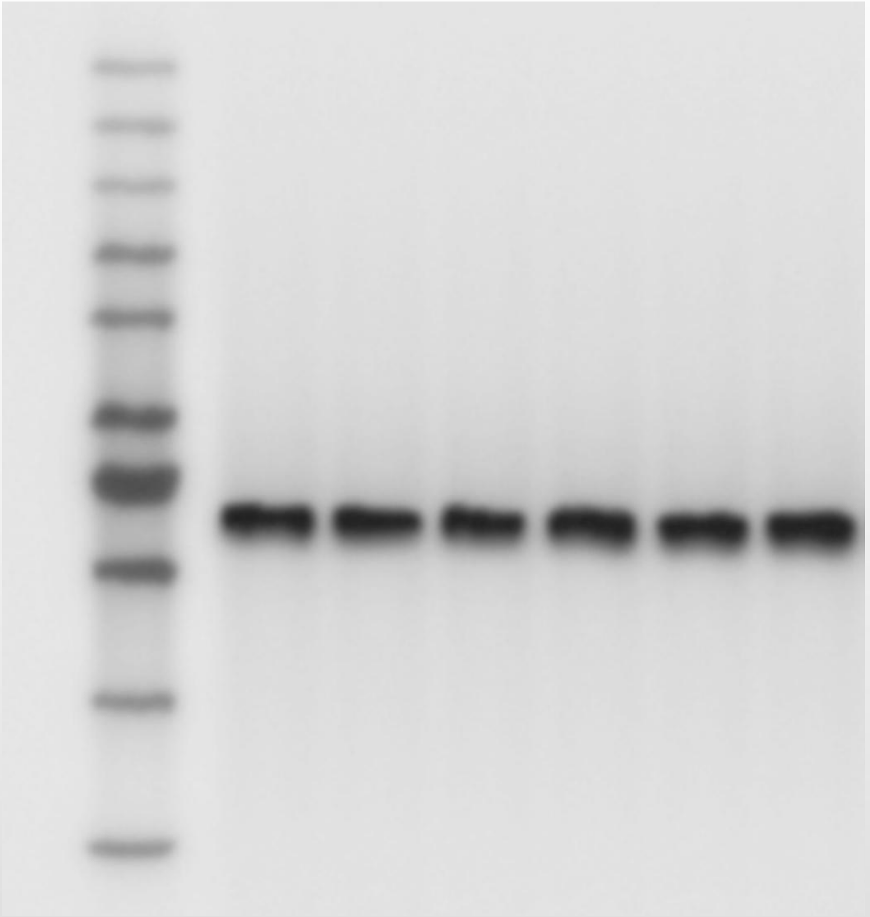

FIGURE6 QA/B/C

A B C

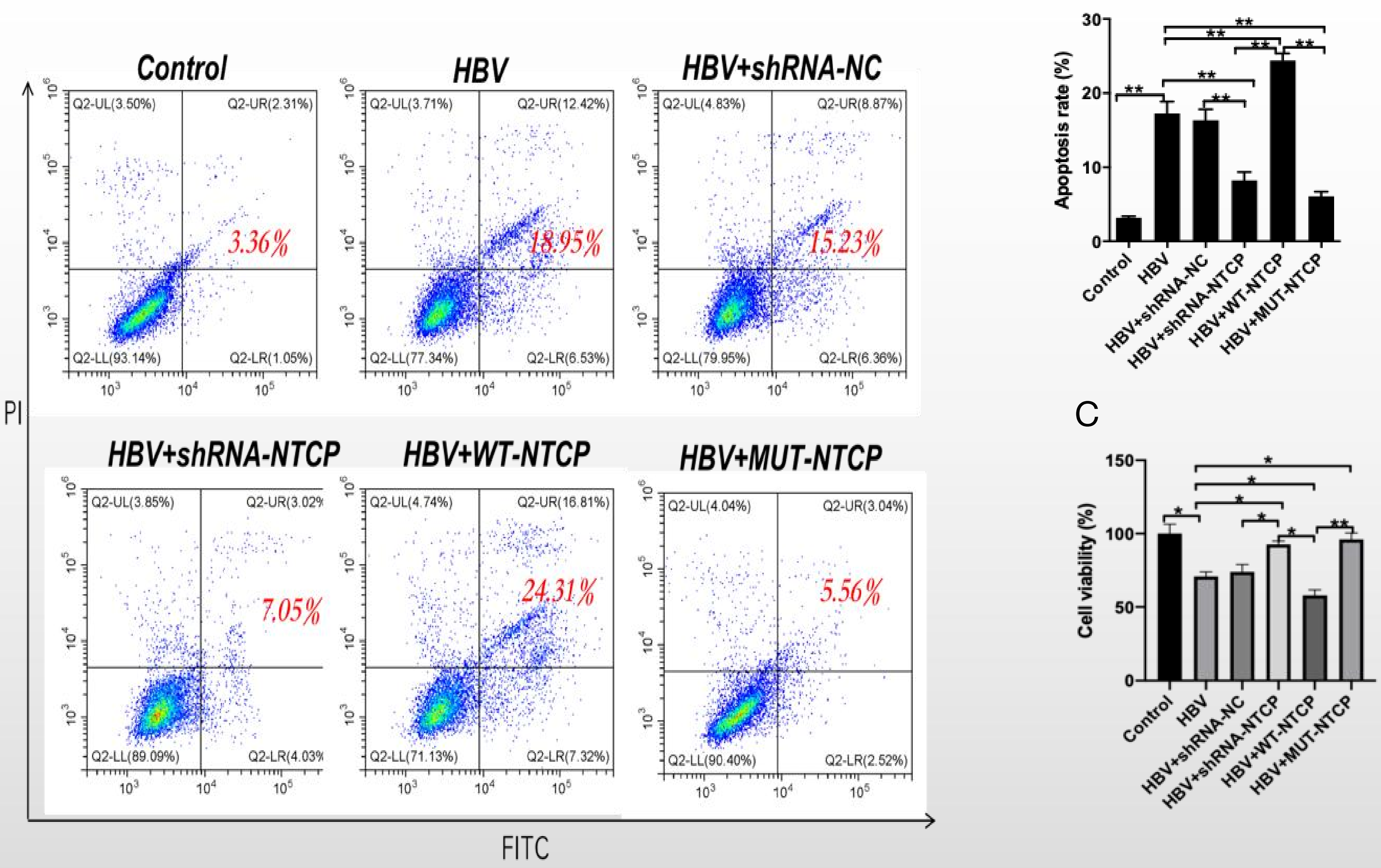

FIGURE6B/C

6B

Control	HBV	HBV+shRNA-NC	HBV+shRNA-NTCP	HBV+WT-NTCP	HBV+MUT-NTCP
Y	Y	Y	Y	Y	Y
3.24	17.13	18.03	8.41	25.43	6.76
2.94	15.77	15.84	7.05	23.58	5.56

6C

Control	HBV	HBV+shRNA-NC	HBV+shRNA-NTCP	HBV+WT-NTCP	HBV+MUT-NTCP
Y	Y	Y	Y	Y	Y
96.86	70.19	78.76	93.79	55.00	100.22
107.38	74.20	68.66	89.99	56.48	91.48
95.76	68.11	74.38	94.21	62.26	96.38

FIGURE7

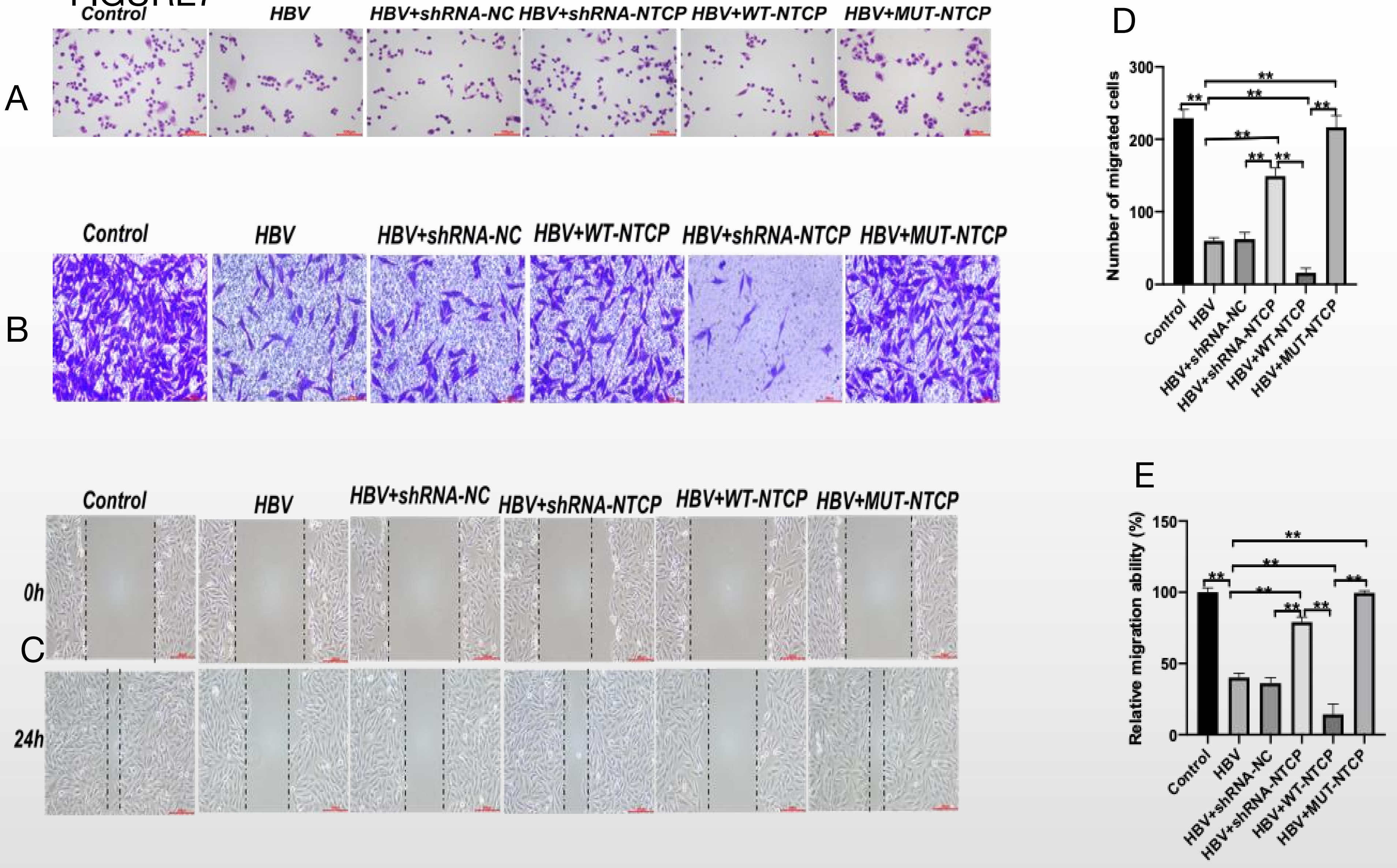
A
Control
HBV
HBV+shRNA-NC
HBV+shRNA-NTCP
HBV+WT-NTCP
HBV+MUT-NTCP
B
Control
HBV
HBV+shRNA-NC
HBV+WT-NTCP
HBV+shRNA-NTCP
HBV+MUT-NTCP
C
Control
HBV
HBV+shRNA-NC
HBV+shRNA-NTCP
HBV+WT-NTCP
HBV+MUT-NTCP
0h
24h
D
Number of migrated cells
0
100
200
300
**
Control
HBV
HBV+shRNA-NC
HBV+shRNA-NTCP
HBV+WT-NTCP
HBV+MUT-NTCP
E
Relative migration ability (%)
0
50
100
150
**
Control
HBV
HBV+shRNA-NC
HBV+shRNA-NTCP
HBV+WT-NTCP
HBV+MUT-NTCP

FIGURE7D/E

7D

Control	HBV	HBV+shRNA-NC	HBV+shRNA-NTCP	HBV+WT-NTCP	HBV+MUT-NTCP
Y	Y	Y	Y	Y	Y
218	61	65	157	23	199
227	64	70	136	15	230
242	55	53	154	10	221

7E

Control	HBV	HBV+shRNA-NC	HBV+shRNA-NTCP	HBV+WT-NTCP	HBV+MUT-NTCP
Y	Y	Y	Y	Y	Y
96.67	36.75	36.70	77.21	7.86	100.77
100.49	41.10	32.18	77.09	12.63	97.78
102.85	42.66	39.83	83.03	22.26	100.04
